# Shifting El Niño inhibits summer Arctic warming and Arctic sea-ice melting over the Canada Basin

**DOI:** 10.1038/ncomms11721

**Published:** 2016-06-02

**Authors:** Chundi Hu, Song Yang, Qigang Wu, Zhenning Li, Junwen Chen, Kaiqiang Deng, Tuantuan Zhang, Chengyang Zhang

**Affiliations:** 1 Institute of Earth Climate and Environment System, Sun Yat-sen University, 135 West Xingang Road, Guangzhou, Guangdong 510275, China; 2 School of Atmospheric Sciences, Nanjing University, Nanjing, Jiangsu 210023, China; 3 School of Atmospheric Sciences, Sun Yat-sen University, Guangzhou, Guangdong 510275, China; 4 Guangdong Province Key Laboratory for Climate Change and Natural Disaster Studies, Sun Yat-sen University, Guangzhou, Guangdong 510275, China; 5 Climate Center, Guangxi Meteorological Bureau, Nanning, Guangxi 530022, China

## Abstract

Arctic climate changes include not only changes in trends and mean states but also strong interannual variations in various fields. Although it is known that tropical-extratropical teleconnection is sensitive to changes in flavours of El Niño, whether Arctic climate variability is linked to El Niño, in particular on interannual timescale, remains unclear. Here we demonstrate for the first time a long-range linkage between central Pacific (CP) El Niño and summer Arctic climate. Observations show that the CP warming related to CP El Niño events deepens the tropospheric Arctic polar vortex and strengthens the circumpolar westerly wind, thereby contributing to inhibiting summer Arctic warming and sea-ice melting. Atmospheric model experiments can generally capture the observed responses of Arctic circulation and robust surface cooling to CP El Niño forcing. We suggest that identification of the equator-Arctic teleconnection, via the ‘atmospheric bridge', can potentially contribute to improving the skill of predicting Arctic climate.

## Abstract (web summary)

Teleconnections between different flavours of El Niño and Arctic climate change are inherently uncertain in the context of warming. Here, the authors show that Central Pacific El Niño contributes to summer Arctic cooling and sea-ice increase via an Equator-Arctic teleconnection on interannual timescales.

## Graphical abstract

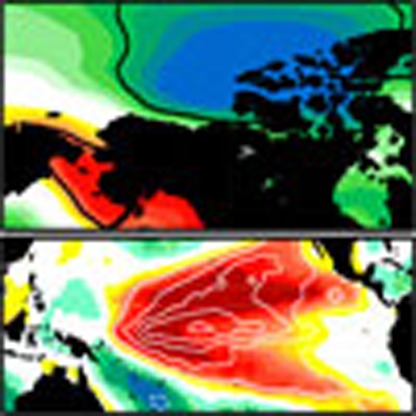

Since the late 1970s, the Arctic Ocean has experienced considerable changes^1^,^2^, such as rapid warming and contracting sea ice. Growing evidence has attributed these changes to the changes in Arctic climate caused by external anthropogenic forcing^3^,^4^,^5^ (that is, increasing aerosol and greenhouse gas emission) and internal natural variability, such as the Atlantic Multidecadal Oscillation^6^, tropical sea surface temperature (SST) trend^7^, as well as anomalous tropical convective heating forcing associated with the El Niño-Southern Oscillation (ENSO)^8^,^9^,^10^ and the Madden–Julian oscillation^11^,^12^,^13^. The effect of Arctic climate change on society is becoming a hot research topic, given the more frequent mid-latitude extreme events because of the increasing Arctic sea-ice loss in the recent decades^14^,^15^,^16^ and the anticipated global changes in future climate scenarios. It is estimated that, by the end of the twenty-first century, the Arctic will probably become an ice-free blue ocean^17^. Many studies have examined Arctic sea-ice responses to externally forced climate changes and found that the region has been warming more quickly than the rest of the world^18^,^19^. However, the ratio of trend to standard deviation of the interannual variability of detrended surface temperature and atmospheric circulation anomalies (Supplementary Fig. 1) is only 10% or so, suggesting that natural interannual variability is dominant and should not be neglected in the context of rapid warming.

Although attributions of the Arctic climate trends to human effects and tropical Pacific SST forcing are now well established, interannual variation of Arctic climate may respond more strongly to the changes in the two prominent types of interannual SST variability in the tropical Pacific, namely, the central Pacific (CP) El Niño and the eastern Pacific (EP) El Niño, both of which are linked to global climate teleconnections^20^,^21^. In the context of global warming, El Niño properties have exhibited considerable changes since the late 1970s, including the changes in frequency, location, intensity and meridional scale^20^,^22^,^23^,^24^. The CP El Niño is occurring more frequently than its counterpart, and tends to become more intense^25^,^26^. On the other hand, the Arctic warming is not spatially uniform but varies significantly with locations^7^,^27^. In addition, the Arctic sea-ice shrinks most strongly during summer and reaches a minimum in September^4^, whereas there is no significant warming trend in the Arctic Ocean in the summer melting season^7^ (see the Extended Data Figure 2 of ref. 7). These seemingly distinct phenomena bring up an intriguing question: Does the almost concurrent regime shift of El Niño play a role in the variability of summer Arctic climate on interannual timescale?

The influence of shifting El Niño on the Arctic climate, compared with the extra-polar climate^21^,^28^,^29^,^30^, however, has received less attention. Projections of the effects of El Niño change on the concurrent Arctic climate change are inherently uncertain. Thus, understanding the impact of shifting El Niño on the Arctic climate is of profound interest.

In this study, we demonstrate that the CP El Niño contributes to Arctic cooling and sea-ice increase during the boreal summer (June–August, JJA) on interannual timescale. Using observational satellite data of summer Arctic sea-ice concentration (SIC) and oceanic/atmospheric reanalysis, together with numerical model simulations, we confirm a teleconnection that exists between the tropical Pacific Ocean and the Arctic Ocean; we also unravel a potential physical mechanism for the teleconnection.

## Results

### Teleconnection between shifting El Niño and Arctic climate

**Figure 1:**
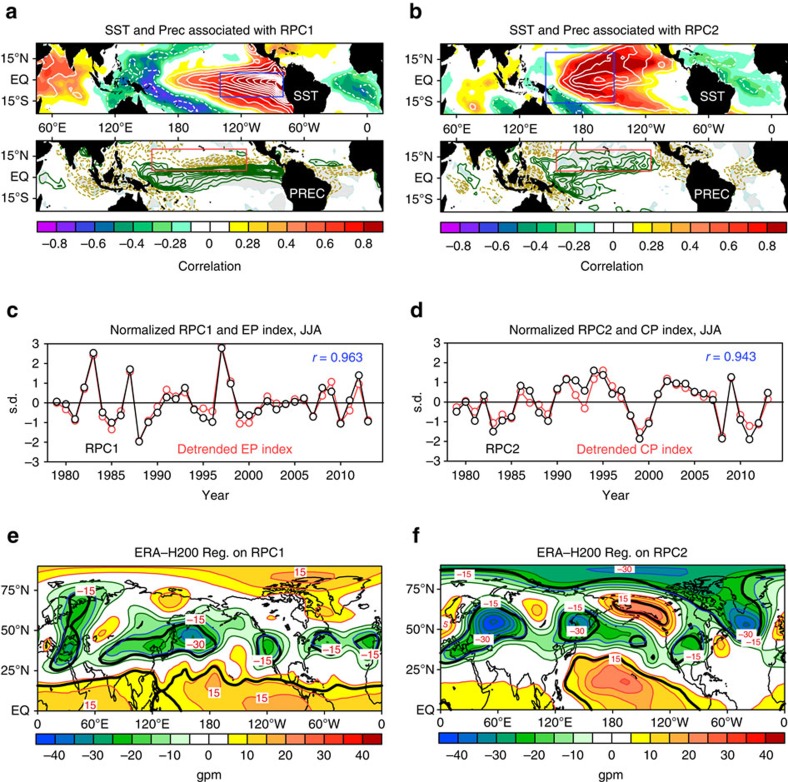
Statistics of EP El Niño and CP El Niño. Spatial patterns of tropical SST: ((**a**) top: colour shadings are used for correlation, and contours are regression with an interval of 0.2 °C) marine precipitation ((**a**) bottom: regression with an interval of 0.4 mm per day) and ERA-H200 ((**e**), regression with an interval of 5 gpm) associated with normalized RPC1 (**c**, in black). Grey shadings and thick black lines indicate the correlation at the estimated 90% confidence level. Panels **b**,**d**,**f** are the same as panels **a**,**c**,**e**, except for RPC2. In **c**,**d**, the time series in red are the detrended and normalized EP and CP indices (see the Methods). Correlation (RPC1, detrended EP index)=0.963, and correlation (RPC2, detrended CP index)=0.943. Blue boxes outline the CP region (140°W–80°W, 12°S–5°N) and the EP region (145°E–150°W, 16°S–20°N), respectively. The two red boxes highlight the same region (155°E–115°W, 5°N–20°N) as a reference system.

The upper panels of Fig. 1a,b show the EP and CP El Niño patterns, respectively, which are derived from a rotated empirical orthogonal function (REOF, see the Methods and Supplementary Note 1 for details and reasons) decomposition of normalized and detrended SST anomalies in a key region (120°E–80°W, 20°S–20°N) of the tropical Pacific. The spatial patterns of REOF1 and REOF2 highlight the positions and magnitudes of SST warming related to the two types of El Niño. As expected, the associated principal components (namely, RPC1 and RPC2) plotted in Fig. 1c,d (black lines) are directly related to detrended EP index and CP index (red lines, see the Methods for details) with large correlation coefficients up to 0.963 and 0.943, respectively. The SST changes related to shifting El Niño lead to changes in convective activity pattern (Fig. 1a,b and Supplementary Fig. 2a,b), which in turn stimulate large-scale Rossby waves via perturbation of upper-level divergence^31^ (Supplementary Fig. 2c). As shown in Fig. 1e,f, the regressions of boreal summer 200-hPa geopotential height (H200) against RPC1 and RPC2 are characterized by planetary-scale atmospheric waves, which result in distant climate anomalies—teleconnections. In comparison with Fig. 1e, Fig. 1f reveals a significant atmospheric teleconnection between CP El Niño and the polar upper troposphere, that is, the significant deepening of tropospheric Arctic polar vortex (APV), in particular over the Canada Basin region, is closely associated with the CP warming (CPW). Similar REOF-related results can be reproduced by a more straightforward way of box-based regression and correlation analysis (Supplementary Figs 3 and 4). The corresponding quasi-geostrophic stream function and Rossby wave activity fluxes (Supplementary Fig. 5) exhibit two distinctive trajectories, which are consistent with the H200 teleconnection patterns (Fig. 1e,f). It is worth noting that the method is independent from the REOF analysis except for the choice of box location, suggesting that the above results are robust and reliable.

**Figure 2:**
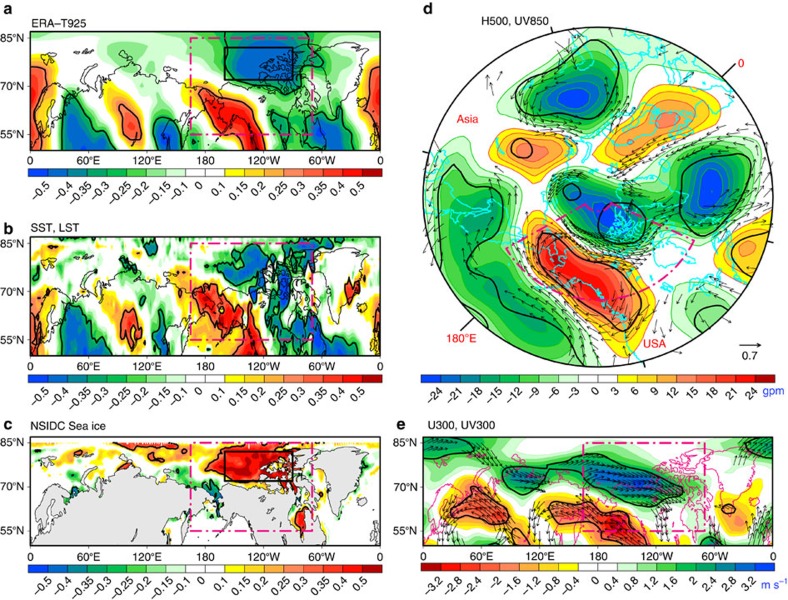
Teleconnection patterns related to central Pacific SST warming. (**a**) Correlations of T925 with RPC2. (**b**,**c**) are the same as **a**, except for SST/LST and SIC, respectively. Shadings in **d**,**e** denote H500 and U300 regressed, respectively, against normalized RPC2. Black arrows in **d**,**e** show the correlations (vectors less than 0.28 omitted) of UV850 and UV300 with RPC2, respectively. Thick black lines indicate correlation at the estimated 90% confidence level. Area-weighted means of T925 and SIC anomalies over the black boxes (160°W–90°W, 72°N–82°N) in **a**,**c** are used to define the T925-index and the SIC-index. The area outlined by the purple dashed box is the same in all five panels. Here, the atmospheric data sets are from the ERA-Interim reanalysis.

Deepening of the APV will inevitably lead to changes in the underlying Arctic climate at surface. As shown in Fig. 2a–c (Supplementary Figs 6 and 7), the regional changes in air temperature at 925-hPa (T925), land/sea surface temperature (LST/SST) and SIC over the Arctic are all significantly correlated with RPC2. The strongest correlation occurs in the Arctic Canada Basin where lower-level northwesterly anomalies (Fig. 2d, vectors; Supplementary Figs 6 and 7) and the resultant cold advection contribute to Arctic cooling and sea-ice increase. Moreover, such response may be amplified by local positive feedbacks (that is, thermal advections and feedbacks of ice-temperature, cloud-radiation and surface sensible/latent heat flux exchanges and so on) among atmosphere, ocean and sea ice^4^,^19^,^32^,^33^, as shown in Supplementary Fig. 8. The 500-hPa geopotential height (H500) anomalies (Fig. 2d, shading; Supplementary Figs 6 and 7) also exhibit a similar wave-train-like feature as that shown in Fig. 1f, accompanied by deepening of the APV and strengthening of the circumpolar westerly wind (CWW) at both lower and upper levels (Fig. 2d,e). In addition, the coherence of lower- and upper-level winds (Fig. 2d,e, vectors) indicates that the circulation anomalies forced by CP El Niño warming are quasi-barotropic. The features linked to RPC1 (Supplementary Fig. 9) are distinct from those associated with RPC2. Motivated by previous studies^7^,^34^,^35^, we thus hypothesize that the CP El Niño plays a key role in forcing the teleconnection pattern that induces the strengthening of CWW and deepening of APV, both of which are conductive to retaining cold air mass in the polar region, favouring regional Arctic cooling and inhibiting Arctic sea-ice melting.

### Identification of key forcing region

To identify the key forcing region in the tropics that determines the wave trains to the Arctic, the combined regression and correlation maps of the tropical Pacific SST, precipitation and Outgoing Longwave Radiation (OLR) anomalies associated with detrended SIC-index and T925-index are shown in Supplementary Fig. 10. The two indices are defined as the area-weighted mean of SIC and T925 anomalies over the region of 160°W–90°W, 72°N–82°N (see the black boxes in Fig. 2a,c, respectively). We can see from the significant areas identified cooperatively by the black boxes in each physical field of SST, precipitation and OLR (Supplementary Fig. 10) that the key forcing region is located in the northern subtropical CP (155°E–115°W, 5°N–20°N). This also indirectly confirms that the interannual variability of Arctic climate anomalies over the Canada Basin is indeed closely linked to the circulation anomalies induced by the convective heating associated with the CP El Niño during summer.

### Convective regimes associated with shifting El Niño

**Figure 3:**
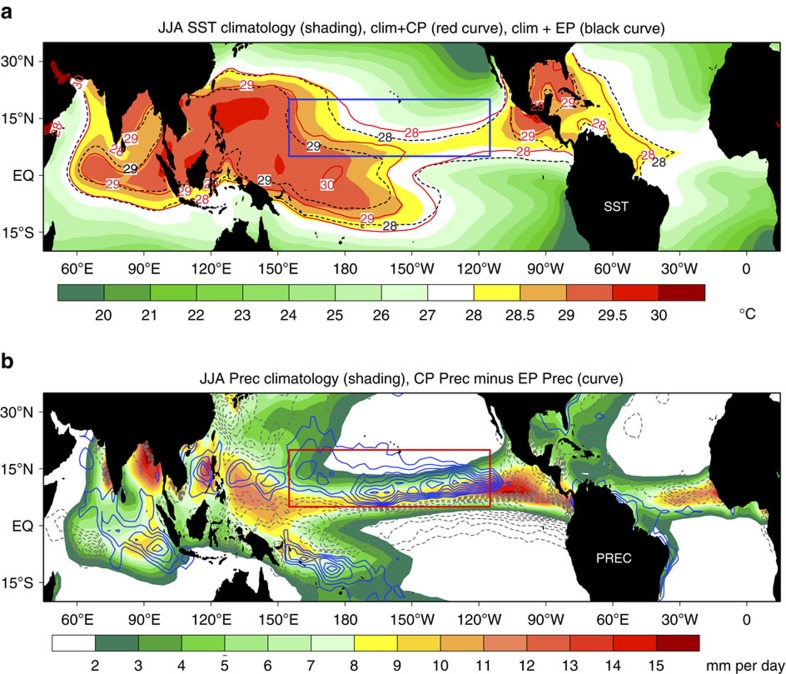
Regime differences between CP El Niño and EP El Niño. (**a**) Composite patterns of total SST for CP El Niño (red solid lines) and EP El Niño (black dashed lines). Total SST is defined as the sum of SST anomalies shown in Fig. 1 plus the SST climatology in the boreal summer (only the total SST above 28 °C is shown, with an interval of 1 °C). (**b**) Composite marine precipitation differences (‘CP minus EP', 0.4 mm per day interval) between CP El Niño and EP El Niño. Shadings in **a**,**b** show the climatology of SST and precipitation, respectively. Blues/red boxes outline the key forcing region (155°E–115°W, 5°N–20°N).

To further elucidate the regime differences in teleconnection patterns associated with the CP and EP El Niño episodes, we present the spatial distributions of total SST (that is, JJA SST climatology plus JJA SST anomalies associated with the CP or EP El Niño) and the composite pattern of precipitation (CP minus EP) in Fig. 3. The isotherms in Fig. 3a indicate the total SST above the warm pool threshold of 28 °C. Note that the black dashed lines (for EP El Niño case) are always surrounded by the red solid lines (for CP El Niño case) over the key forcing region (155°E–115°W, 5°N–20°N; see box in Fig. 3). In other words, during the CP El Niño, the 28 °C isotherm (north of the equator) extends into the white-shading areas where the JJA SST climatology is below 28 °C, which reinforces the climatological SST over the key forcing region and favours the poleward shift and strengthening of the inter-tropical convergence zone (ITCZ). Correspondingly, the enhanced convective precipitation along the ITCZ and its northern side over the key forcing region (Fig. 3b) confirms the northward shift of the intensifying ITCZ. Thus, the potential physical mechanism for the CPW-Arctic teleconnection could be understood as follows. A poleward shift of enhanced ITCZ, which indicates a poleward shift of enhanced tropical convection forcing, will inevitably lead to poleward shifts of upper-level divergence^31^, northern flank of the Hadley cell, and the subtropical jet^36^,^37^, reinforcing the poleward propagating Rossby waves^36^,^38^ and favouring a further northward location of large-scale teleconnection linked more easily to the polar region.

### Modelling support from NCAR CAM4

To further assess the causal relationship between CPW forcing and Arctic circulation response, numerical simulations are performed using the US National Center for Atmospheric Research (NCAR) global Community Atmosphere Model version 4 (CAM4; see the Methods for details). The simulated Arctic H200 response (Supplementary Fig. 11a) to CPW is significantly negative and comparable to the observed H200 pattern over the Arctic Ocean (Fig. 1f), suggesting that the model is capable of reproducing the deepening of the APV, in spite of some excursions in its position and magnitude. The CAM4 also well reproduces the observed strengthening of CWW and Arctic cooling (Supplementary Fig. 11b,c), implying that the observed Arctic cooling and SIC increase are the consequence of changes in upper-level circulation driven by tropical CPW to a certain extent. Thus, the declining H200 and widespread cooling over the Arctic Ocean can be viewed as an integral of linear responses to the imposed tropical SST anomalies related to the CP El Niño.

Although the simulated Arctic cooling is robust, the simulated position and magnitude of Arctic climate responses are not stationary, leading to some excursions in various composite patterns of the CAM4 results (Supplementary Fig. 11). Moreover, the simulated responses do not match the REOF results completely and therefore should be interpreted carefully. The difference is possibly because (i) atmospheric circulation and temperature are also affected by the changes in SIC, snow cover/depth and others; and (ii) inevitable difference exists between the observed and simulated climatologies of the background flow and temperature and so on. Therefore, the total response induced by the changes in atmospheric circulation may depend on snow–atmosphere–SIC–SST interactions, which are not fully included in the model. Nonetheless, the simulated results suggest that the deepening APV associated with CP El Niño warming contributes to the regional Arctic cooling.

### Case study

**Figure 4:**
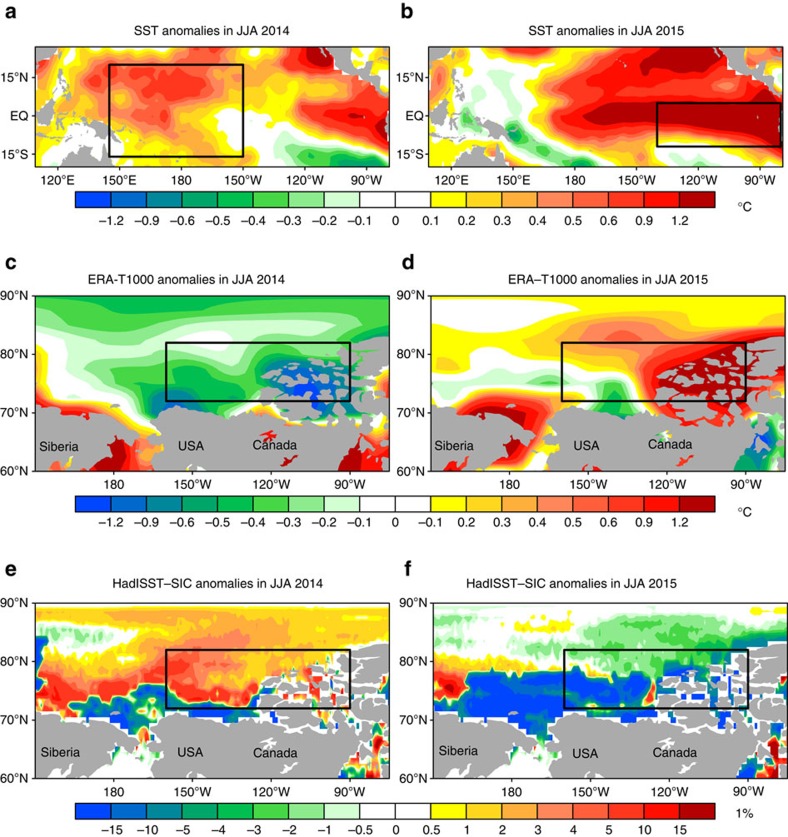
Case verifications in JJA of 2014 and 2015. Shown in (**a**,**c**,**e**) are the SST, 1,000-hPa air temperature (ERA-Interim) and HadISST SIC anomalies in JJA of 2014, respectively. (**b**,**d**,**f**) are the same as in **a**,**c**,**e**, except for JJA of 2015. The boxes in **a**,**b** outline the CP region (140°W–80°W, 12°S–5°N) and the EP region (145°E–150°W, 16°S–20°N), respectively. The boxes in **c**–**f** mark the same region (160°W–90°W, 72°N–82°N).

The above empirical findings are directly derived from the data sets for 1979–2013. If shifting El Niño really contributes to Arctic cooling, there should be less Arctic warming and less sea-ice melting in JJA of 2014 (CP El Niño) and more Arctic warming and more sea-ice melting in JJA of 2015 (EP El Niño; see http://www.emc.ncep.noaa.gov/research/cmb/sst_analysis/images/archive/monthly_anomaly/). For verification, we show these two latest cases in Fig. 4 (and Supplementary Fig. 12). The tropical Pacific SST anomalies during JJA in 2014 and in 2015 mirror an episode of robust CP El Niño and a typical event of EP El Niño (Fig. 4a,b), respectively. As shown in Fig. 4c–f, the corresponding phases of low-level air temperature and SIC anomalies over the Arctic Canada Basin also exhibit highly consistent one-to-one relationship to the two types of El Niño in the two summers, respectively. Thus, the two contrasting cases provide further support that shifting El Niño contributes to inhibiting summer Arctic warming and Arctic sea-ice melting over the Canada Basin.

## Discussion

This study demonstrates a linkage between CPW and summer Arctic climate from the perspective of detrended interannual variability. Our straightforward analyses of the tropical Pacific SST and corresponding precipitation anomalies reveal the following features. First, the linear response of upper-level circulation to CP El Niño warming is robust over the Arctic polar region, whereas the response to EP El Niño warming is not apparent although the intensity of EP El Niño is approximately twice of that of CP El Niño. Second, the discrepancy in the effects of two types of El Niño on the Arctic circulation can be explained by the background of higher SST over the CP than over the EP, favouring deeper convection along the ITCZ and to its north. Poleward shift of the enhanced ITCZ favours a further northward location of large-scale teleconnection to the Arctic region. Third, the teleconnection induced by the CP El Niño can cause deepening and strengthening APV and CWW. Finally, the changes in the Arctic circulation in turn provide favourable large-scale dynamic conditions for cold air mass to be retained in the polar region, leading to Arctic cooling and sea-ice increase, especially over the Canada Basin. Two more points should be emphasized: (i) observed results are consistent with those reproduced based on different EP and CP El Niño definitions; (ii) the results derived from the detrended CP index are most robust as the CP index contains more signals, such as the warming trend that is not well captured by other CP El Niño definitions (see Supplementary Note 2, Supplementary Tables 1 and 2 and Supplementary Figs 13–16 for more details).

**Figure 5:**
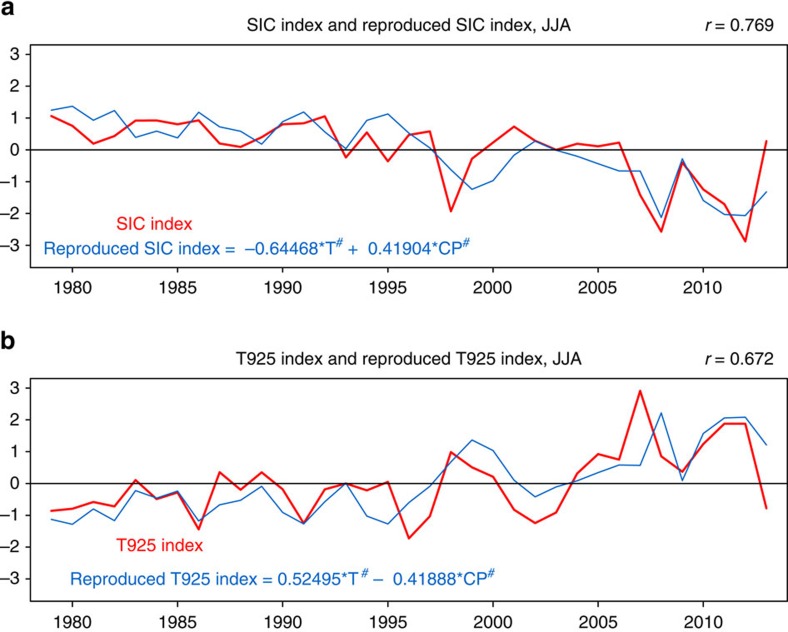
Original and reproduced time series of SIC-index and T925-index. (**a**) The normalized SIC-index and the reproduced SIC-index, as well as their correlation. (**b**) The normalized T925-index and the reproduced T925-index, as well as their correlation. The reproduced indices are derived from the multiple regression equations as shown in the figure (in the two equations, ‘T^#^' and ‘CP^#^' indicate the normalized trend time series and the normalized and detrended CP index, respectively).

Hence, the CP El Niño contributes to inhibiting summertime Arctic warming and sea-ice loss on interannual timescale to a certain extent. In other words, the summer Arctic sea-ice loss would be more intense without the effect of more frequent CP El Niño events over the past few decades (Fig. 5). It is clear that the regression coefficients of ‘T^#^' and ‘CP^#'^ in the two equations shown in Fig. 5 are comparable but opposite with each other, suggesting that both the linear trend and the interannual CP El Niño affect the climate variability over the Arctic Canada Basin, at least for the recent decades. However, it may be just a matter of time before external forcing dominates the Arctic warming because of the foreseeable sustainable increase in greenhouse gases and aerosols^27^. Our study highlights one of the often-overlooked aspects of Arctic climate responses to the anticipated change in El Niño events on interannual timescale in the context of global warming. On the other hand, Kim *et al*.^28^ has demonstrated that the predictability of CPW (for example, CP El Niño) has no obvious spring barrier that exists in the case of EP El Niño^39^. Therefore, as shown in Supplementary Fig. 17, the CP El Niño-Arctic teleconnection potentially enhances our understanding of the predictability of the changes in summer Arctic climate under the greenhouse warming background.

We note that our argument for summer is different from that in the winter situation—EP El Niño can lead to winter Arctic cooling via a tropically excited Arctic warming mechanism^8^. However, there is no evidence indicating that the EP El Niño can also lead to summer Arctic cooling via the tropically excited Arctic warming mechanism. It is seen that the seasonal evolutions of Canada Basin surface temperature associated with the CP and EP indices are different from each other (Supplementary Fig. 18), although the features are similar among various seasons for both CP El Niño patterns and EP El Niño patterns. Different teleconnection mechanisms between summer and winter seem to be due mainly to seasonal reversal of climatological background state and to different air–sea interactions within and/or outside the Pacific Ocean^40^, that is, different amplitudes and locations of the warm pool^41^, ITCZ/heating and the basic flow^37^ (Supplementary Fig. 19). For example, evidence has indicated that the ENSO modulates the polar vortex mainly by the Aleutian low via the Pacific-North American pattern during the boreal winter^37^,^42^,^43^, but this mechanism seems less relevant in summer. This argument is consistent with that of Sung *et al*.^44^, which suggested that the differences in polar responses between CP El Niño and EP El Niño arose from variation of seasonal evolution of extratropical waves, which depends strongly on the source location of tropical heating. Accordingly, here we emphasize that the summer teleconnections associated with CP and EP El Niño events are substantially distinct from the winter teleconnections^8^,^45^ because of the differences in background climate.

We also note that the response of winter stratospheric polar vortex to the ENSO is sensitive to the phase of Quasi-biannual Oscillation^43^,^46^,^47^ and to the stratospheric sudden warming^48^. Other factors such as the Arctic sea ice^49^, Eurasian snow^50^, extra-polar SST trend^51^ and volcanic eruption^52^ can also strongly affect the winter stratospheric polar vortex. Whether the response of boreal summer tropospheric APV to CP El Niño also depends on a third factor (for example, sea ice, snow, Madden–Julian oscillation, Atlantic Multidecadal Oscillation and so on) is a subject of further investigation. Nonetheless, the respective role of each factor has not been systematically examined, albeit these factors may be interconnected. In short, the changes in Arctic climate (that is, air temperature, sea ice, APV, CWW and so on) are influenced by many factors, including atmospheric circulation, surface heat flux, ocean heat transport, ocean temperature, ice-albedo feedback, atmospheric CO_2_ and surface air temperature and so on. The respective roles and their sensitivities to tropical SST forcing of these factors may be different between winter and summer. The CPW-Arctic teleconnection proposed here provides an addition to the possible factors that affect tropospheric APV and Arctic climate during the boreal summer.

## Methods

### Observational data sets

The averages of two SST data sets obtained from the NOAA Extended Reconstructed SST version 3b^53^ (ERSSTv3b; http://www.esrl.noaa.gov/psd/data/gridded/data.noaa.ersst.html) and the Hadley Centre Global Sea Ice and Sea Surface Temperature^54^ (HadISST; http://www.metoffice.gov.uk/hadobs/hadisst/data/download.html) are used. The HadISST SST is interpolated onto the same 2° × 2° longitude–latitude grid as the NOAA SST. Also used are the averages of two precipitation data sets, namely, the Global Precipitation Climatology Project version 2.2^55^ (http://www.esrl.noaa.gov/psd/data/gridded/data.gpcp.html) and the Climate Prediction Center Merged Analysis of Precipitation^56^ (http://www.esrl.noaa.gov/psd/data/gridded/data.cmap.html). The NOAA Interpolated OLR^57^ (http://www.esrl.noaa.gov/psd/data/gridded/data.interp_OLR.html), the NSIDC SIC^58^ (http://nsidc.org/data/docs/daac/nsidc0079_bootstrap_seaice.gd.html) from the Nimbus-7 SMMR and DMSP SSM/I-SSMIS Version 2, the HadISST SIC (http://www.metoffice.gov.uk/hadobs/hadisst/data/download.html) and the COBE^59^ SIC (http://www.esrl.noaa.gov/psd/data/gridded/data.cobe2.html) are also used. The ERA-Interim reanalysis^60^ (http://apps.ecmwf.int/datasets/) is provided by the European Centre for Medium-Range Weather Forecasts, and the MERRA reanalysis^61^ (http://disc.sci.gsfc.nasa.gov/daac-bin/DataHoldings.pl) by the Global Modeling and Assimilation Office at NASA Goddard Space Flight Center. The JRA-55 reanalysis^62^ (http://jra.kishou.go.jp/JRA-55/index_en.html) is provided by the Climate Prediction Division, Global Environment and Marine Department, of the Japan Meteorological Agency. In addition to the reanalysis temperatures, combined observed air/marine temperature anomalies are obtained from (i) the NASA Goddard Institute for Space Studies surface temperature analysis^63^ (http://www.esrl.noaa.gov/psd/data/gridded/data.gistemp.html) and (ii) the HadCRUT4 data set^64^ (http://www.esrl.noaa.gov/psd/data/gridded/data.crutem4.html). Also used is the NOAA GHCN_CAMS 2 m LST^65^ (http://www.esrl.noaa.gov/psd/data/gridded/data.ghcncams.html). For detection of interannual variation, all data sets are first normalized and detrended, unless noted otherwise. The base period used in this study is 1979–2013.

### Analysis methodology and significance test

As suggested by Garfinkel *et al*.^45^ attentions are needed when selecting indices/methods to identify the CP El Niño. Accordingly, following Lian and Chen^66^, in this study we apply the method of REOF decomposition^67^ (see Supplementary Note 1 for details and reasons) to identify the dominant modes (namely, the two types of El Niño) of tropical Pacific SST anomalies (120°E–80°W, 20°S–20°N). All regression results are shown by the values of regression coefficient scaled by a factor of 2. Correspondingly, a value of correlation (*r*) above 0.28 is used to estimate the 90% confidence level for a 35-year length of 1979–2013. Here, *r* is defined as


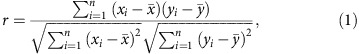


where 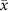 and 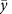 are the sample mean values of *x*_*i*_ and *y*_*i*_ from *i=1* to *i=n* (*n* is the sample size). For composite analysis, we determine the statistical significance level based on the Student's *t*-test. Here, *t* is defined as


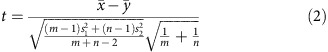


where 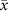 and 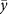 are the sample mean values of *x*_*i*_ from *i=1* to *i=m* and *y*_*i*_ from *i=1* to *i=n* (*m* and *n* are the sample sizes); 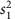 and 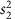 are the sample variances; 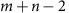 is the degrees of freedom.

### EP index and CP index

The detrended EP and CP indices are individually defined as the area mean of normalized and detrended SST anomalies at each grid in the regions marked by the blue boxes shown in Fig. 1a (140°W–80°W, 12°S–5°N) and Fig. 1b (145°E–150°W, 16°S–20°N), respectively; and the resulted indices are further normalized to obtain a unit s.d.

### Rossby wave activity flux

We use the Rossby wave activity analysis^68^,^69^ to reveal quasi-stationary Rossby wave propagations. The analysis is suitable for detecting the propagating characteristics of large-scale quasi-stationary planetary waves.

### NCAR climate model

To further confirm the CPW-Arctic teleconnection and establish the causal relationship between CP El Niño SST forcing and Arctic regional climate anomalies, experiments are performed with the global CAM4 (ref. 70) from the US NCAR. The CAM4 is the atmospheric component of the Community Earth System Model (CESM 1.2.0), with a 1.9° × 2.5° finite volume grid at 26 hybrid sigma levels. The atmospheric composition is kept at the values of year 2000 (for example, the CO_2_ concentration is set as a constant 367.0 p.p.m. in the simulations). A 40-year control run is forced with annually cycled climatological monthly mean SST (1979–2013). The forced run is similar to the control run, but is forced by the total SST constructed by adding the climatological SST and the SST anomalies associated with the CP El Niño in each calendar month. The SST anomalies are constructed by regressing SST anomalies over the tropical region (40°E–80°W, 25°S–25°N) to the CP index and then scaling it by a factor of 2. The impacts produced by CP El Niño warming in the forced run are identified by subtracting the results from the control run. For a more comprehensive comparison, the Arctic responses during the corresponding successive 10 years (that is, the 31–40 years), 20 years (that is, the 21–40 years) and 30 years (that is, the 11–40 years) are used to investigate the model responses to specified SST forcing.

### CAM4 code availability

The CAM4 code is available via the URL: https://svn-ccsm-models.cgd.ucar.edu/cesm1/release_tags/cesm1_2_2/.

### Data availability

All raw data can be accessed via the links provided above.

## Additional information

**How to cite this article:** Hu, C. *et al*. Shifting El Niño inhibits summer Arctic warming and Arctic sea-ice melting over the Canada Basin. *Nat. Commun.* 7:11721 doi: 10.1038/ncomms11721 (2016).

## Supplementary Material

Supplementary Information Supplementary Figures 1-19, Supplementary Tables 1-2, Supplementary Notes 1-2 and Supplementary References.

## References

[b1] Duarte C., Lenton T., Wadhams P. & Wassmann P. Abrupt climate change in the Arctic. Nat. Clim. Change 2, 60–62 (2012).

[b2] Jeffries M., Overland J. & Perovich D. The Arctic shifts to a new normal. Phys. Today 66, 35–40 (2013).

[b3] Gillett N. P. . Attribution of polar warming to human influence. Nat. Geosci 1, 750–754 (2008).

[b4] Serreze M. C., Barrett A. P., Stroeve J. C., Kindig D. N. & Holland M. M. The emergence of surface-based Arctic amplification. Cryosphere 3, 11–19 (2009).

[b5] Polyakov I., Walsh J. E. & Kwok R. Recent changes of Arctic multiyear sea ice coverage and the likely causes. Bull. Am. Meteorol. Soc 93, 145–151 (2012).

[b6] Chylek P., Folland C. K., Lesins G., Dubey M. K. & Wang M. Y. Arctic air temperature change amplification and the Atlantic multidecadal oscillation. Geophys. Res. Lett. 36, L14801 (2009).

[b7] Ding Q. . Tropical forcing of the recent rapid Arctic warming in northeastern Canada and Greenland. Nature 509, 209–212 (2014). 24805345 10.1038/nature13260

[b8] Lee S. Testing of the tropically excited Arctic warming mechanism (TEAM) with traditional El Niño and La Niña. J. Clim 25, 4015–4022 (2012).

[b9] Lee S., Feldstein S. B., Pollard D. & White T. S. Do planetary wave dynamics contribute to equable climates? J. Clim 24, 2391–2404 (2011).

[b10] Lee S., Gong T., Johnson N., Feldstein S. & Pollard D. On the possible link between tropical convection and the Northern Hemisphere Arctic surface air temperature change between 1958 and 2001. J. Clim 24, 4350–4367 (2011).

[b11] Yoo C., Feldstein S. & Lee S. The impact of the Madden–Julian oscillation trend on the Arctic amplification of surface air temperature during the 1979–2008 boreal winter. Geophys. Res. Lett. 38, L24804 (2011).

[b12] Yoo C., Lee S. & Feldstein S. B. Arctic response to an MJO-like tropical heating in an idealized GCM. J. Atmos. Sci. 69, 2379–2393 (2012).

[b13] Yoo C., Lee S. & Feldstein S. B. Mechanisms of Arctic surface air temperature change in response to the Madden–Julian oscillation. J. Clim 25, 5777–5790 (2012).

[b14] Screen J. A. Influence of Arctic sea ice on European summer precipitation. Environ. Res. Lett. 8, 044015 (2013).

[b15] Tang Q., Zhang X., Yang X. & Francis J. A. Cold winter extremes in northern continents linked to Arctic sea ice loss. Environ. Res. Lett. 8, 014036 (2013).

[b16] Tang Q., Zhang X., Yang X. & Francis J. A. Extreme summer weather in northern mid-latitudes linked to a vanishing cryosphere. Nat. Clim. Change 4, 45–50 (2014).

[b17] Boé J., Hall A. & Qu X. September sea ice cover in the Arctic Ocean projected to vanish by 2100. Nat. Geosci 2, 341–343 (2009).

[b18] Graversen R. G., Mauritsen T., Tjernström M., Källén E. & Svensson G. Vertical structure of recent Arctic warming. Nature 451, 53–56 (2008). 18172495 10.1038/nature06502

[b19] Screen J. A. & Simmonds I. The central role of diminishing sea ice in recent Arctic temperature amplification. Nature 464, 1334–1337 (2010). 20428168 10.1038/nature09051

[b20] Yeh S.-W., Kug J.-S., Dewitte B., Kwon M.-H., Kirtman B. P. & Jin F.-F. El Niño in a changing climate. Nature 461, 511–514 (2009). 19779449 10.1038/nature08316

[b21] Ashok K., Behera S. K., Rao S. A., Weng H. & Yamagata T. El Niño Modoki and its possible teleconnection. J. Geophys. Res. 112, C11007 (2007).

[b22] An S.-I. & Wang B. Interdecadal change of the structure of the ENSO mode and its impact on the ENSO frequency. J. Clim 13, 2044–2055 (2000).

[b23] McPhaden M. J., Zebiak S. E. & Glantz M. H. ENSO as an integrating concept in Earth science. Science 314, 1740–1745 (2006). 17170296 10.1126/science.1132588

[b24] Zhang W., Li J. & Jin F.-F. Spatial and temporal features of ENSO meridional scales. Geophys. Res. Lett. 36, L15605 (2009).

[b25] Lee T. & McPhaden M. J. Increasing intensity of El Niño in the central-equatorial Pacific. Geophys. Res. Lett. 37, L14603 (2010).

[b26] Yu J.-Y. & Kim S. T. Identifying the types of major El Niño events since 1870. Int. J. Climatol. 33, 2105–2113 (2013).

[b27] Bader J. Climate science: the origin of regional Arctic warming. Nature 509, 167–168 (2014). 24805339 10.1038/509167a

[b28] Kim H.-M., Webster P. J. & Curry J. A. Impact of shifting patterns of Pacific Ocean warming on North Atlantic tropical cyclones. Science 325, 77–80 (2009). 19574388 10.1126/science.1174062

[b29] Yu J.-Y. & Zou Y. The enhanced drying effect of Central-Pacific El Niño on US winter. Environ. Res. Lett. 8, 014019 (2013).

[b30] Karori M. A., Li J. & Jin F.-F. The asymmetric influence of the two types of El Niño and La Niña on summer rainfall over southeast China. J. Clim 26, 4567–4582 (2013).

[b31] Sardeshmukh P. D. & Hoskins B. J. The generation of global rotational flow by steady idealized tropical divergence. J. Atmos. Sci. 45, 1228–1251 (1988).

[b32] Kay J. E., L'Ecuyer T., Gettelman A., Stephens G. & O'Dell C. The contribution of cloud and radiation anomalies to the 2007 Arctic sea ice extent minimum. Geophys. Res. Lett. 35, L08503 (2008).

[b33] Ogi M. & Wallace J. M. The role of summer surface wind anomalies in the summer Arctic sea ice extent in 2010 and 2011. Geophys. Res. Lett. 39, L09704 (2012).

[b34] Li X., Holland D. M., Gerber E. P. & Yoo C. Impacts of the north and tropical Atlantic Ocean on the Antarctic Peninsula and sea ice. Nature 505, 538–542 (2014). 24451542 10.1038/nature12945

[b35] Graf H.-F. & Zanchettin D. Central Pacific El Niño, the ‘‘subtropical bridge,'' and Eurasian climate. J. Geophys. Res. 117, D01102 (2012).

[b36] Hoskins B. J. & Karoly D. J. The steady linear response of a spherical atmosphere to thermal and orographic forcing. J. Atmos. Sci. 38, 1179–1196 (1981).

[b37] Jin F. & Hoskins B. J. The direct response to tropical heating in a baroclinic atmosphere. J. Atmos. Sci. 52, 307–319 (1995).

[b38] Lee S. & Yoo C. On the causal relationship between poleward heat flux and the Equator-to-Pole temperature gradient: a cautionary tale. J. Clim. 27, 6519–6525 (2014).

[b39] Webster P. J. & Yang S. Monsoon and ENSO: selectively interactive systems. Quart. J. Roy. Meteor. Soc. 118, 877–926 (1992).

[b40] Alexander M. A. . The atmospheric bridge: the influence of ENSO teleconnections on air–sea interaction over the global oceans. J. Clim. 15, 2205–2231 (2002).

[b41] Wang B., Wu R. & Fu X. Pacific–East Asian teleconnection: How does ENSO affect East Asian climate? J. Clim. 13, 1517–1536 (2000).

[b42] Horel J. D. & Wallace J. M. Planetary-scale atmospheric phenomena associated with the Southern Oscillation. Mon. Wea. Rev. 109, 813–829 (1981).

[b43] Garfinkel C. I. & Hartmann D. L. Different ENSO teleconnections and their effects on the stratospheric polar vortex. J. Geophys. Res. 113, D18114 (2008).

[b44] Sung M.-K., Kim B.-M. & An S.-I. Altered atmospheric responses to eastern Pacific and central Pacific El Niños over the North Atlantic region due to stratospheric interference. Clim. Dyn. 42, 159–170 (2014).

[b45] Garfinkel C. I., Hurwitz M. M., Waugh D. W. & Butler A. H. Are the teleconnections of central Pacific and eastern Pacific El Niño distinct in boreal wintertime? Clim. Dyn. 41, 1835–1852 (2013).

[b46] Garfinkel C. I. & Hartmann D. L. Effects of the El Niño southern oscillation and the Quasi-Biennial oscillation on polar temperatures in the stratosphere. J. Geophys. Res. 112, D19112 (2007).

[b47] Garfinkel C. I., Hartmann D. L. & Sassi F. Tropospheric precursors of anomalous Northern Hemisphere stratospheric polar vortices. J. Clim. 23, 3282–3299 (2010).

[b48] Iza M. & Calvo N. Role of stratospheric sudden warmings on the response to Central Pacific El Niño. Geophys. Res. Lett. 42, 2482–2489 (2015).

[b49] Kim B.-M. . Weakening of the stratospheric polar vortex by Arctic sea-ice loss. Nat. Commun. 5, 4646 (2014). 25181390 10.1038/ncomms5646

[b50] Cohen J., Barlow M., Kushner P. J. & Saito K. Stratosphere–troposphere coupling and links with Eurasian land surface variability. J. Clim. 20, 5335–5343 (2007).

[b51] Garfinkel C. I., Hurwitz M. M. & Oman L. D. Effect of recent sea surface temperature trends on the Arctic stratospheric vortex. J. Geophys. Res. Atmos. 120, 5404–5416 (2015).

[b52] Graf H.-F., Zanchettin D., Timmreck C. & Bittner M. Observational constraints on the tropospheric and near-surface winter signature of the Northern Hemisphere stratospheric polar vortex. Clim. Dyn. 43, 3245–3266 (2014).

[b53] Smith T. M., Reynolds R. W., Peterson T. C. & Lawrimore J. Improvements to NOAA's historical merged land–ocean surface temperature analysis (1880–2006). J. Clim. 21, 2283–2296 (2008).

[b54] Rayner N. A. . Global analyses of sea surface temperature, sea ice, and night marine air temperature since the late nineteenth century. J. Geophys. Res. 108, 4407 (2003).

[b55] Adler R. F. . The Version-2 Global Precipitation Climatology Project (GPCP) monthly precipitation analysis (1979–present). J. Hydrometeor 4, 1147–1167 (2003).

[b56] Xie P. & Arkin P. A. Global precipitation: A 17-year monthly analysis based on gauge observations, satellite estimates, and numerical model outputs. Bull. Amer. Meteor. Soc. 78, 2539–2558 (1997).

[b57] Liebmann B. & Smith C. A. Description of a complete (Interpolated) Outgoing Longwave Radiation dataset. Bull. Am. Meteorol. Soc. 77, 1275–1277 (1996).

[b58] Comiso J. C. Updated 2015. Bootstrap Sea Ice Concentrations from Nimbus-7 SMMR and DMSP SSM/I-SSMIS Version 2. Boulder, Colorado USA: NASA National Snow and Ice Data Center Distributed Active Archive Center http://dx.doi.org/10.5067/J6JQLS9EJ5HU (2000).

[b59] Hirahara S., Ishii M. & Fukuda Y. Centennial-scale sea surface temperature analysis and its uncertainty. J. Clim. 27, 57–75 (2014).

[b60] Dee D. P. . The ERA-Interim reanalysis: configuration and performance of the data assimilation system. Quat. J. R. Meteorol. Soc. 137, 553–597 (2011).

[b61] Rienecker M. M. . MERRA: NASA's modern-era retrospective analysis for research and applications. J. Clim. 24, 3624–3648 (2011). 10.1175/JCLI-D-16-0758.1 PMC6999672 32020988

[b62] Kobayashi S. . The JRA-55 Reanalysis: general specifications and basic characteristics. J. Meteor. Soc. Jpn. 93, 5–48 (2015).

[b63] Hansen J., Ruedy R., Sato M. & Lo K. Global surface temperature change. Rev. Geophys. 48, RG4004 (2010).

[b64] Morice C. P., Kennedy J. J., Rayner N. A. & Jones P. D. Quantifying uncertainties in global and regional temperature change using an ensemble of observational estimates: The HadCRUT4 dataset. J. Geophys. Res. 117, D08101 (2012).

[b65] Fan Y. & van den Dool H. A global monthly land surface air temperature analysis for 1948–present. J. Geophys. Res. 113, D01103 (2008).

[b66] Lian T. & Chen D. An evaluation of rotated EOF analysis and its application to tropical pacific SST variability. J. Clim 25, 5361–5373 (2012).

[b67] Horel J. D. A rotated principal component analysis of the interannual variability of the Northern Hemisphere 500-mb height field. Mon. Wea. Rev. 109, 2080–2092 (1981).

[b68] Plumb R. A. On the three-dimensional propagation of stationary waves. J. Atmos. Sci. 42, 217–229 (1985).

[b69] Takaya K. & Nakamura H. A formulation of a phase-independent wave-activity flux for stationary and migratory quasigeostrophic eddies on a zonally varying basic flow. J. Atmos. Sci. 58, 608–627 (2001).

[b70] Neale R. B. . The mean climate of the Community Atmosphere Model (CAM4) in forced SST and fully coupled experiments. J. Clim. 26, 5150–5168 (2013).

