# Supplementary material for: Shifting El Niño inhibits summer Arctic warming and Arctic sea-ice melting over the Canada Basin
Source: Nat Commun. 2016 Jun 2;7:11721. doi: 10.1038/ncomms11721 (PMC4895717; doi:10.1038/ncomms11721)
Supplement: Supplementary Information — Supplementary Figures 1-19, Supplementary Tables 1-2, Supplementary Notes 1-2 and Supplementary References. [file ncomms11721-s1.pdf]

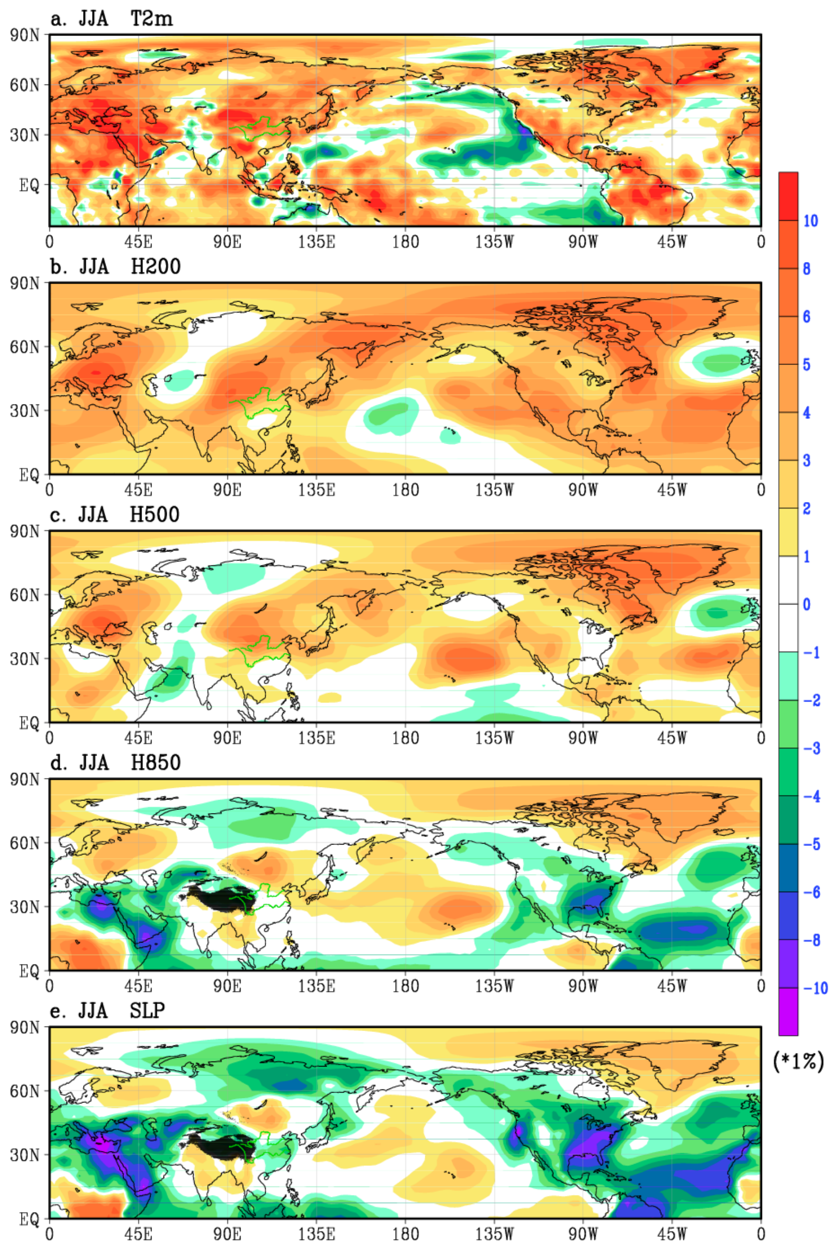

Supplementary Figure 1 | Ratio of trend to standard deviation of detrended interannual variability for 1979–2013. Shown in **a** is the 2-m temperature (T2m); Shown in **b–e** are same as **a**, except for H200, H500, H850 and SLP, respectively, which are from the ERA-Interim reanalysis.

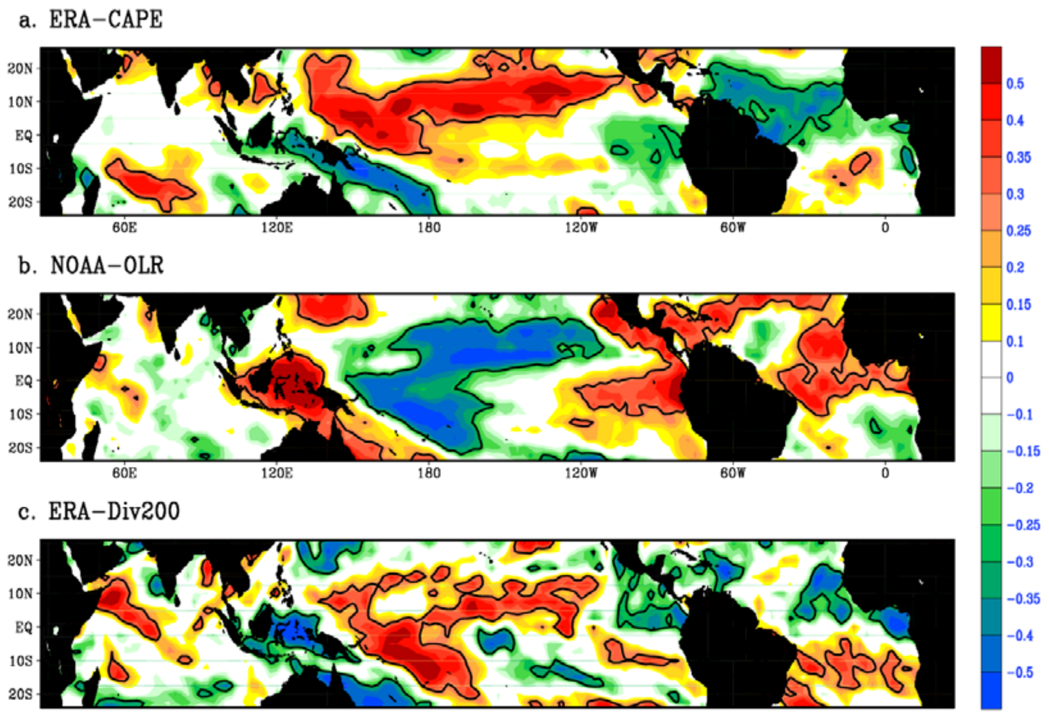

Supplementary Figure 2 | Convective activity patterns. (**a**) Correlations of convective available potential energy with CP El Niño (RPC2). Shown in **b** and **c** are the same as **a**, except for NOAA OLR and 200-hPa ERA divergence. Thick black lines indicate the correlation at the estimated 90% confidence level.

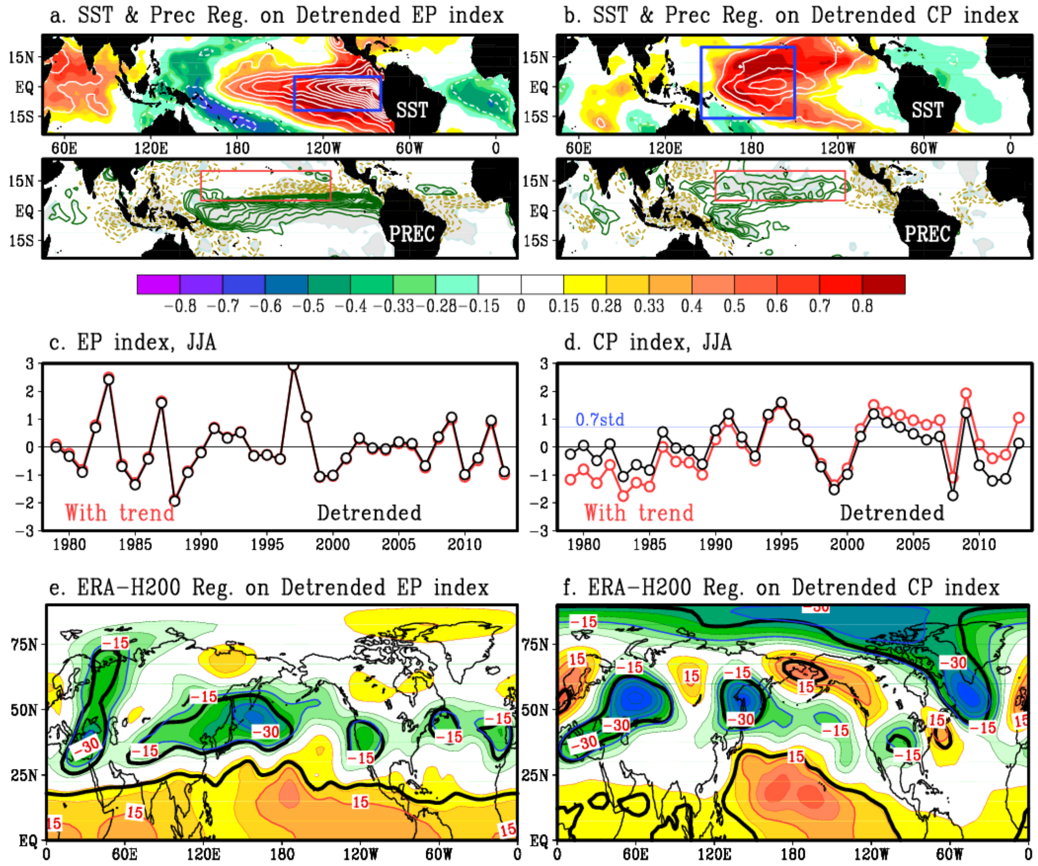

Supplementary Figure 3 | Statistics of detrended EP index and CP index. Spatial patterns of tropical SST (**a**, top: color shadings are used for correlation, and contours are regression with an interval of 0.2°C), marine precipitation (**a**, bottom: regression with an interval of 0.4 mm d^{-1}), and ERA-H200 (**e**, regression with an interval of 5 gpm) associated with the detrended EP index (**c**, in black). Gray shadings and thick black lines indicate the correlation at the estimated 90% confidence level. Panels **b**, **d** and **f** are the same as panels **a**, **c** and **e**, except for the detrended CP index. Note that the red time series in **c** and **d** are the EP index and the CP index with trend, respectively. The thin line shown in **d** indicates the 0.7 standard deviations (0.7 s.d.). There are twelve CP El Niño summers (criteria: 0.7 s.d.) such as 1991, 1994, 1995, 1996, 2002, 2003, 2004, 2005, 2006, 2007, 2009, and 2013, mostly corresponding to the CP El Niño winters identified by Iza & Calvo (ref. 10, see their Table 1). Blue boxes outline the CP region (140°W–80°W, 12°S–5°N) and the EP region (145°E–150°W, 16°S–20°N), respectively. The two red boxes are the same (155°E–115°W, 5°N–20°N).

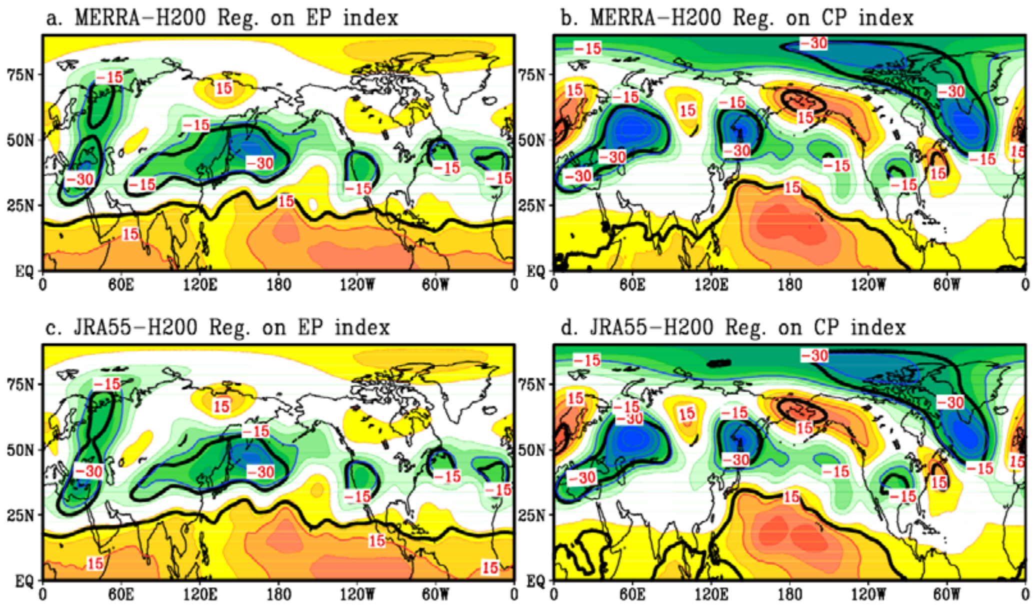

Supplementary Figure 4 | Regressions of H200 anomalies onto the detrended EP index and CP index. Spatial patterns of MERRA-H200 (regression with an interval of 5 gpm) associated with (**a**) the detrended EP index and (**b**) the detrended CP index. Thick black lines indicate the correlation at the estimated 90% confidence level. Panels **c–d** are the same as **a–b**, except for the JRA55-H200.

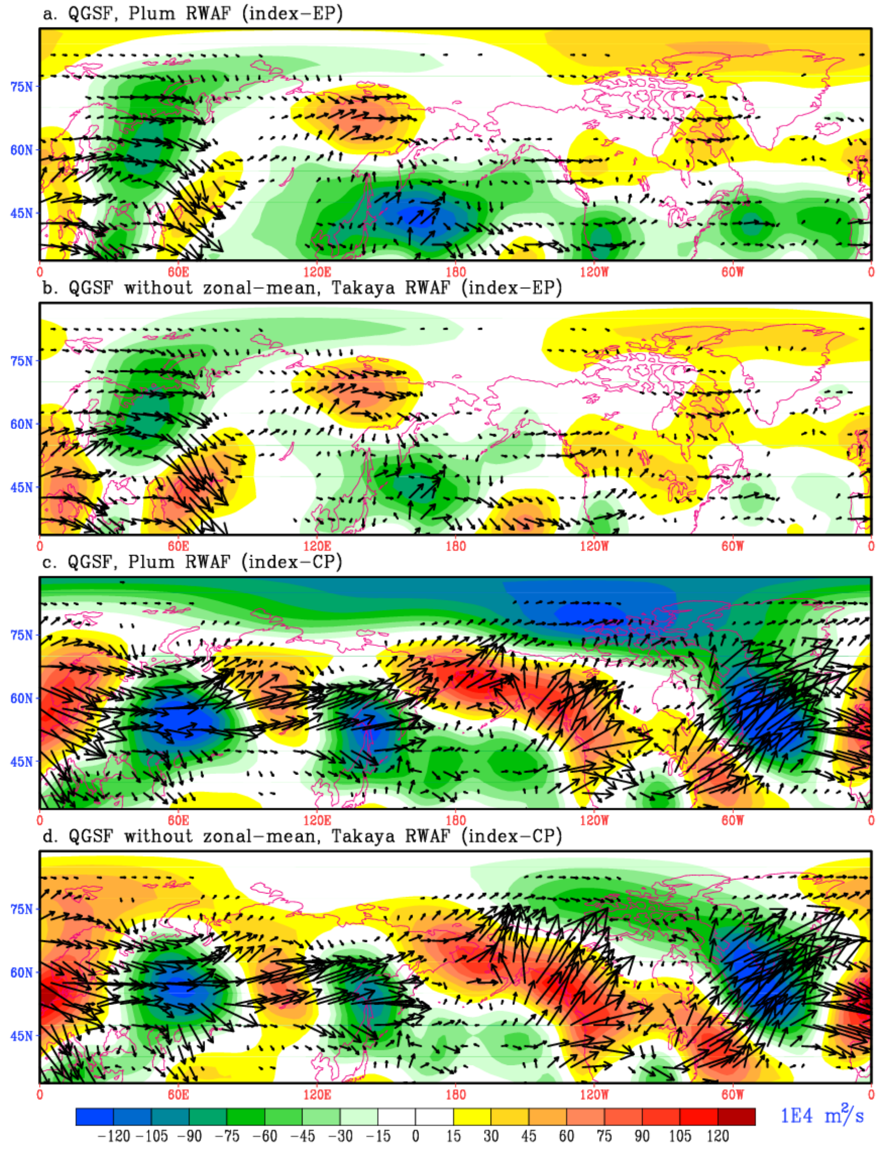

Supplementary Figure 5 | Quasi-geostrophic stream function and stationary Rossby wave activity flux. Panels **a** and **b** are associated with the detrended EP index. (**a**) Shadings (units: 10^4 m^2 s^{-1}) and arrows (units: 10^6 m^2 s^{-2}, vectors less than 10^5 m^2 s^{-2} omitted) indicate the stream function and the wave activity flux defined by Plumb (ref. 15), respectively. Shown in **b** is the same as **a**, except for the stream function without zonal mean and the wave activity flux defined by Takaya and Nakamura (ref. 16). Panels **c** and **d** are the same as **a** and **b**, except for the detrended CP index. Here the stream function and the wave activity flux are determined in terms of the mean of 500-hPa and 300-hPa data (ERA-Interim reanalysis).

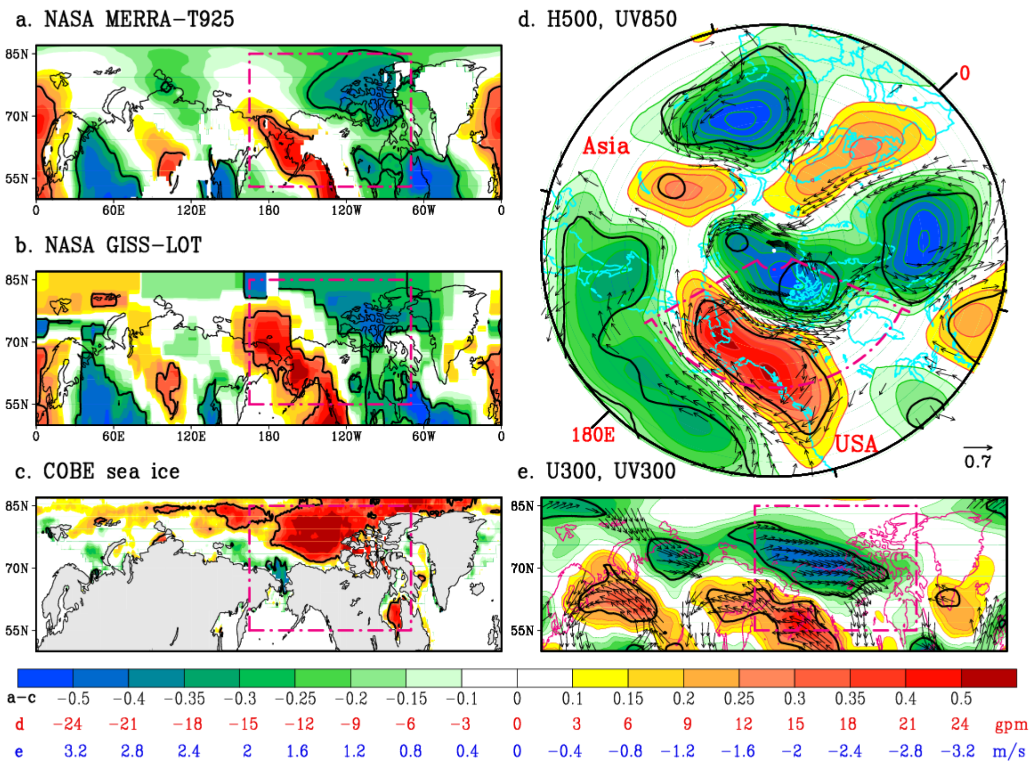

Supplementary Figure 6 | Teleconnection patterns related to CP SST warming. (**a**) Correlations of MERRA-T925 with RPC2. Panels **b** and **c** are the same as **a**, except for GISS-LOT and COBE-SIC, respectively. Shadings in **d** and **e** denote MERRA-H500 and MERRA-U300, regressed respectively against normalized RPC2. Black arrows in **d** and **e** show the correlations (vectors less than 0.28 omitted) of MERRA-UV850 and MERRA-UV300 with RPC2, respectively. Thick black lines indicate correlation at the estimated 90% confidence level. The area outlined by the purple dashed box is the same in all five panels.

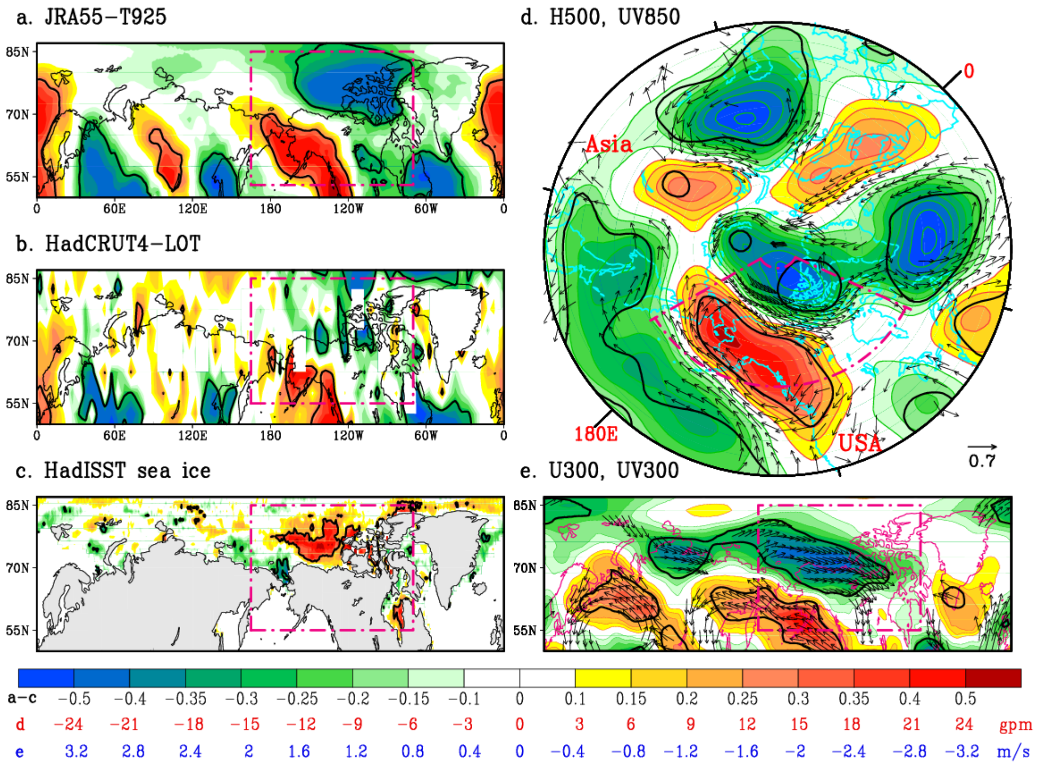

Supplementary Figure 7 | Teleconnection patterns related to CP SST warming. (**a**) Correlations of JRA55-T925 with RPC2. Panels **b** and **c** are the same as **a**, except for HadCRUT4-LOT and HadISST-SIC, respectively. Shadings in **d** and **e** denote JRA55-H500 and JRA55-U300, regressed respectively against normalized RPC2. Black arrows in **d** and **e** show the correlations (vectors less than 0.28 omitted) of JRA55-UV850 and JRA55-UV300 with RPC2, respectively. Thick black lines indicate correlation at the estimated 90% confidence level. The area outlined by the purple dashed box is the same in all five panels.

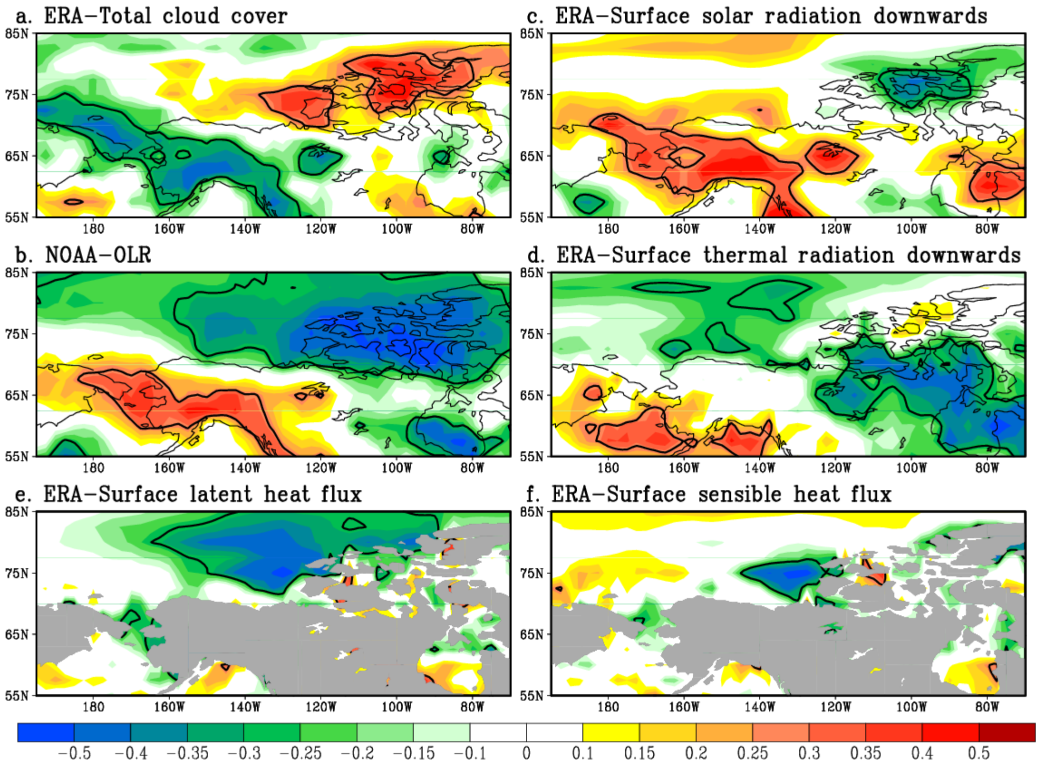

Supplementary Figure 8 | Local feedbacks. (**a**) Correlations of total cloud cover with CP El Niño (RPC2). Panels **b**–**f** are the same as **a**, except for NOAA OLR, ERA downward surface solar radiation, ERA downward surface thermal radiation, and ERA surface latent and sensible heat flux. Thick black lines indicate the correlation at the estimated 90% confidence level.

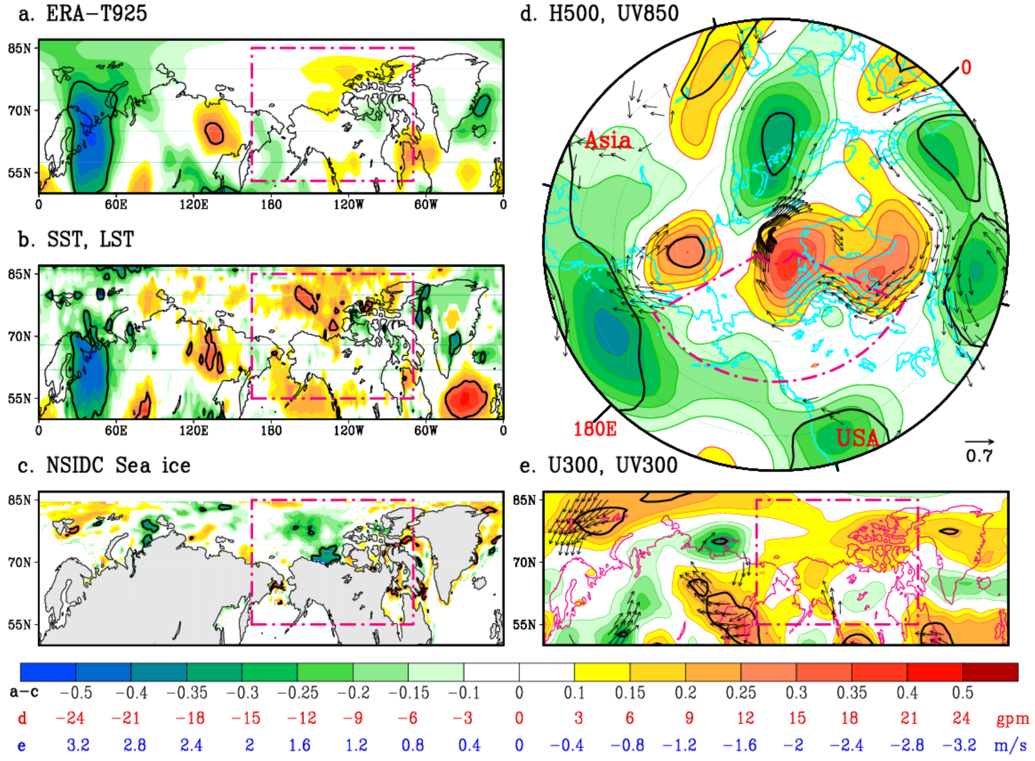

Supplementary Figure 9 | Teleconnection patterns related to EP SST warming. (**a**) Correlations of T925 with RPC1. Panels **b** and **c** are the same as **a**, except for SST/LST and NSIDC-SIC, respectively. Shadings in **d** and **e** denote H500 and U300, regressed respectively against normalized RPC1. Black arrows in **d** and **e** show the correlations (vectors less than 0.28 omitted) of UV850 and UV300 with RPC1, respectively. Thick black lines indicate correlation at the estimated 90% confidence level. Here, the atmospheric data sets are from the ERA-Interim reanalysis. The area outlined by the purple dashed box is the same region in all five panels. There is no Arctic cooling mode over the Canada Basin but a weak warming instead.

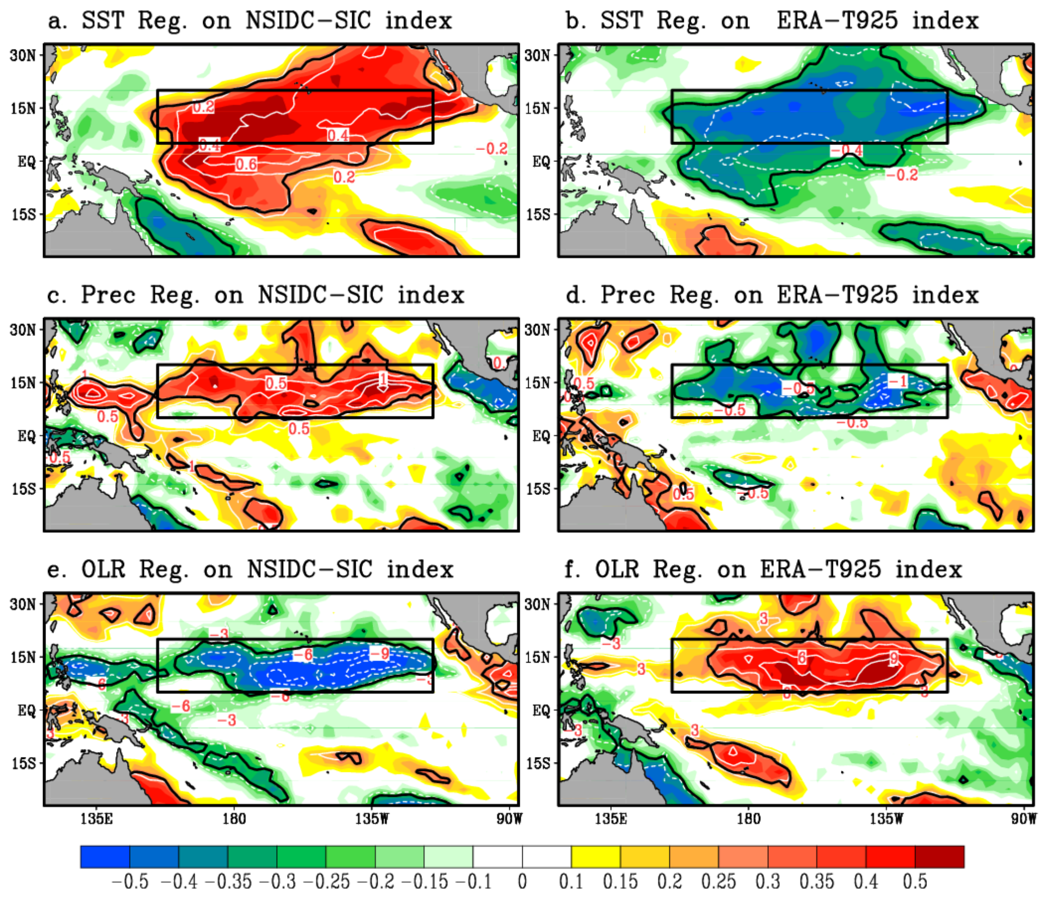

Supplementary Figure 10 | Identification of key forcing regions. (**a**–**b**) Correlations (shading) and regressions (contour) of tropical Pacific SST with the detrended SIC-index and the detrended T925-index (see the black boxes in Fig. 2a and Fig. 2c), respectively. Panels **c**–**f** are the same as **a**–**b**, except for precipitation (**c**–**d**) and OLR (**e**–**f**). Thick black lines indicate the correlation at the estimated 90% confidence level. The significant areas cooperatively identified by the black boxes in each physical filed (SST, precipitation, and OLR) shed light on that the key forcing region is located over the region (155°E–115°W, 5°N–20°N).

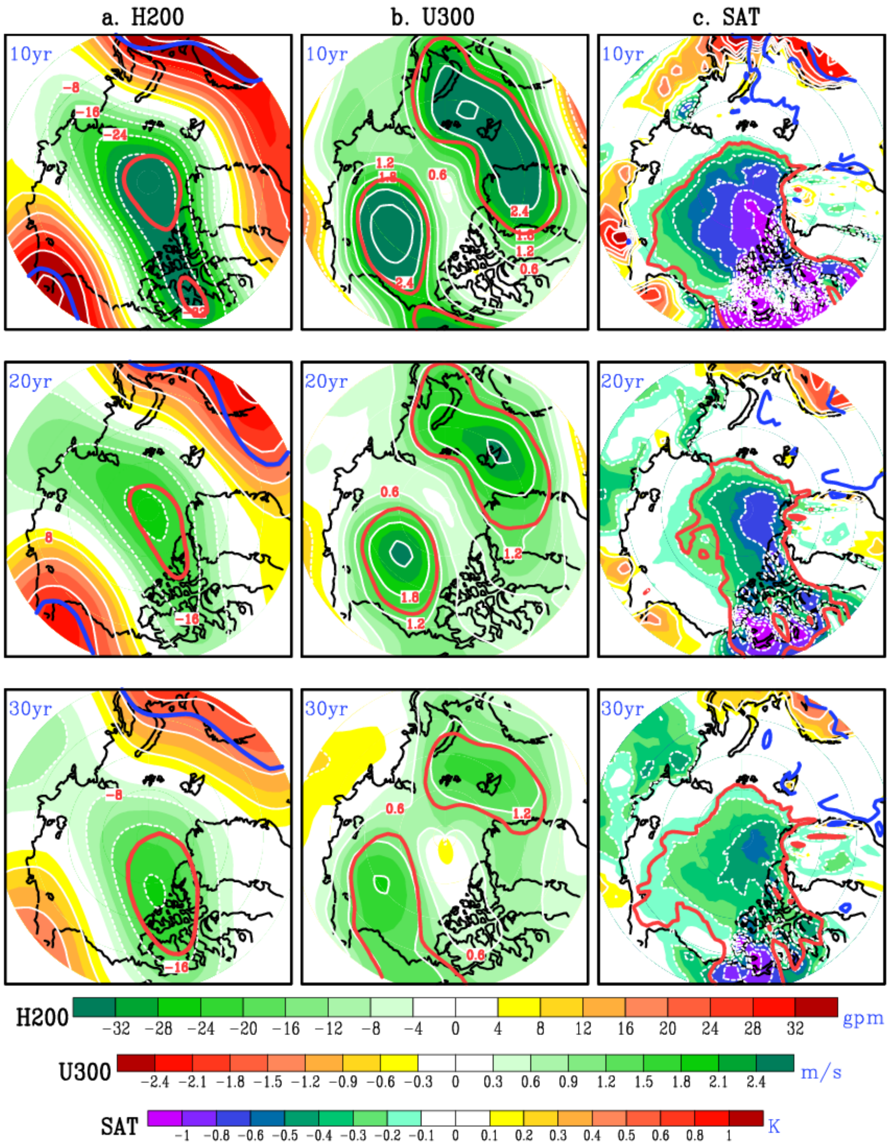

Supplementary Figure 11 | CAM4 simulated summer Arctic response to CP El Niño forcing. (**a**) H200, (**b**) U300, and (**c**) SAT. Upper, middle, and lower panels show the Arctic responses during the corresponding successive 10 years (i.e., the 31–40 years), 20 years (i.e., the 21–40 years) and 30 years (i.e., the 11–40 years) between the control run and the forcing run, respectively. (**a**) H200 and (**b**) U300 exhibit a response of deepened Arctic polar vortex and strengthened circumpolar westerly wind, implying that the CPW plays a key role in the Equator-Arctic teleconnection. All three responses show similar mechanism as in observed results over the Arctic, in spite of some drifts/excursions in amplitude and position. Thick lines indicate the areas where the composite differences in H200, U300, and SAT between the forcing run and the control run are statistically at the estimated 90% confidence level by the *t*-test (composite analysis, see **Methods**).

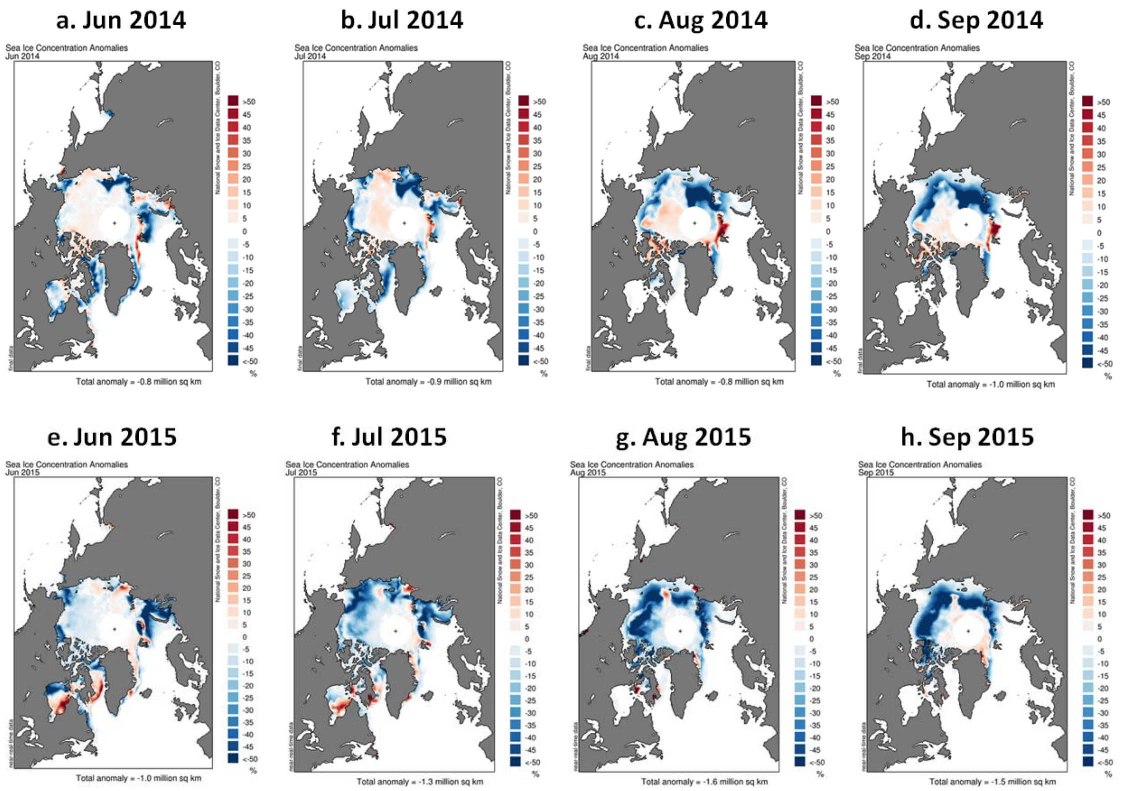

Supplementary Figure 12 | Case verifications. Monthly NSIDC-SIC anomalies in summer of 2014 (CP El Niño) and summer of 2015 (EP El Niño), downloaded from the National Snow & Ice Data Center (http://nsidc.org/data/seaice_index/archives/image_select.html). It is clear that there was less Arctic sea-ice loss in summer of 2014 (CP El Niño) and more Arctic sea-ice melting in summer of 2015 (EP El Niño) over the Canada Basin, supporting our argument about the CPW-Arctic cooling teleconnection.

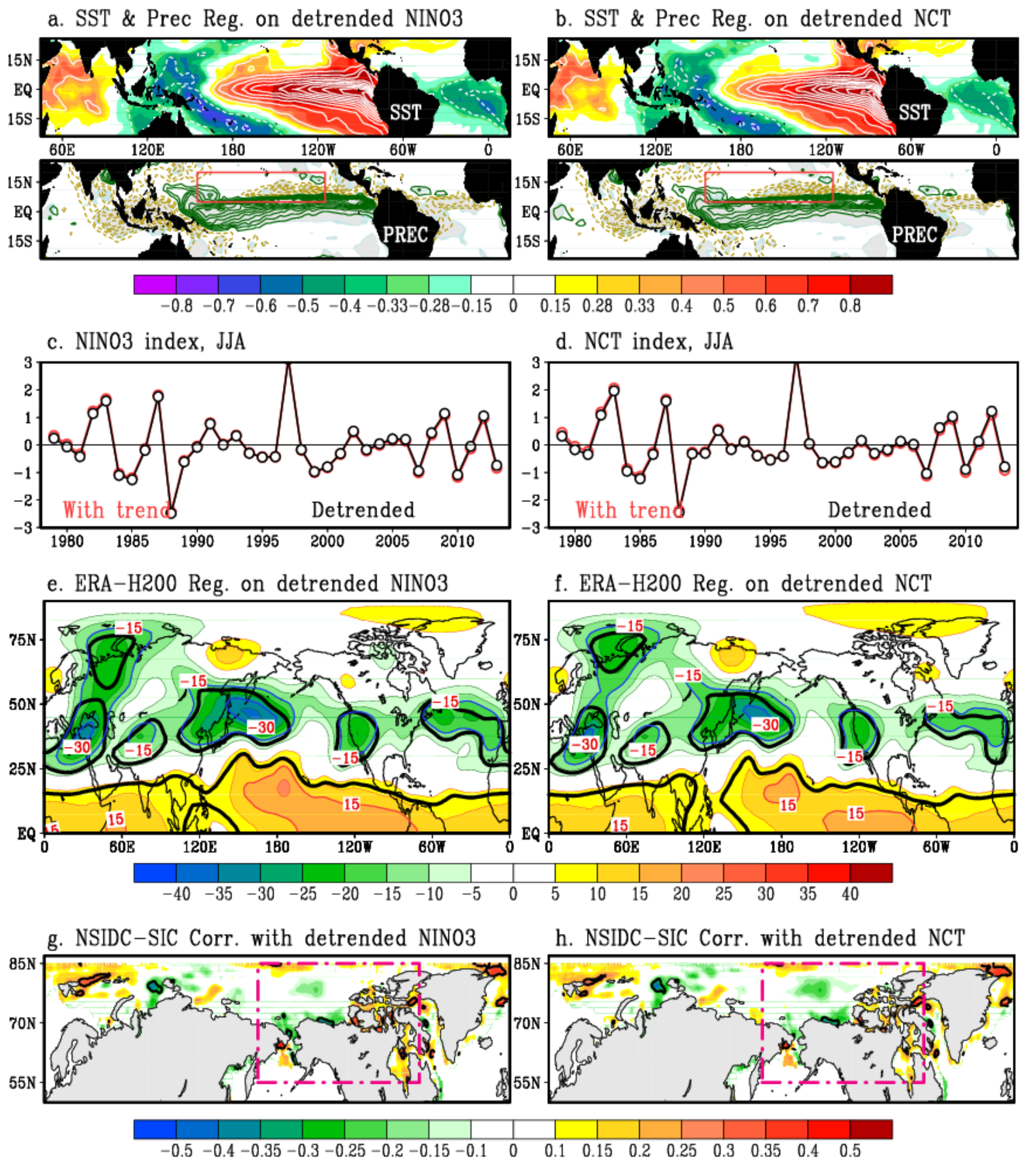

Supplementary Figure 13 | Statistics of detrended Niño3 and NCT indices. Spatial patterns of tropical SST (**a**, top: color shadings are used for correlation, and contours are regression with an interval of 0.2°C), marine precipitation (**a**, bottom: regression with an interval of 0.4 mm day^{-1}), ERA-H200 (**e**, regression with an interval of 5 gpm) and NSIDC-SIC (**g**, correlation) associated with the detrended Niño3 index (**c**, in black). Gray shadings and thick black lines indicate the correlation at the estimated 90% confidence level. Panels **b**, **d**, **f** and **h** are the same as panels **a**, **c**, **e** and **g**, except for the detrended NCT index. Note that the red time series in **c** and **d** are the Niño3 index and the NCT index with trend.

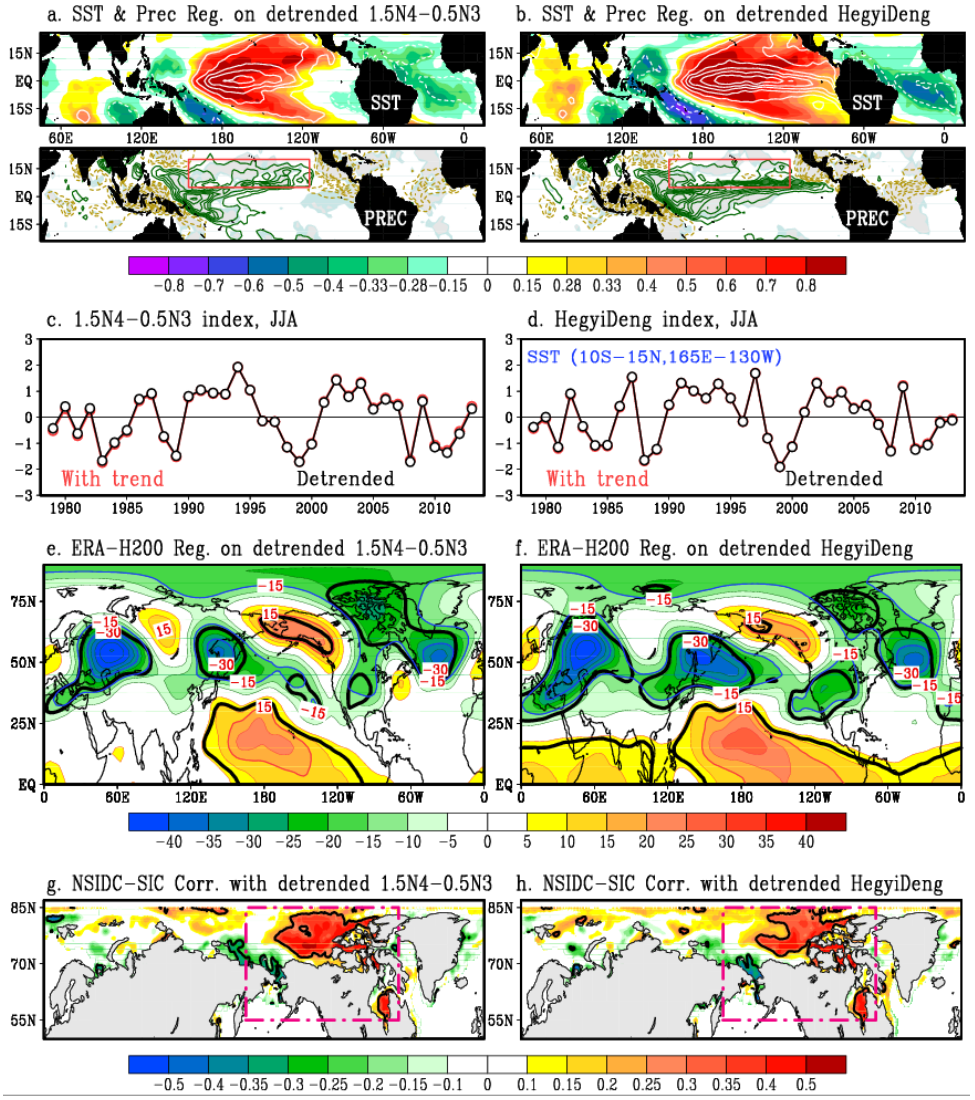

Supplementary Figure 14 | Statistics of detrended 1.5N4-0.5N3 and HegyiDeng indices. Spatial patterns of tropical SST (**a**, top: color shadings are used for correlation, and contours are regression with an interval of 0.2°C), marine precipitation (**a**, bottom: regression with an interval of 0.4 mm day^{-1}), ERA-H200 (**e**, regression with an interval of 5 gpm) and NSIDC-SIC (**g**, correlation) associated with the detrended 1.5N4-0.5N3 index (**c**, in black). Gray shadings and thick black lines indicate the correlation at the estimated 90% confidence level. Panels **b**, **d**, **f** and **h** are the same as panels **a**, **c**, **e** and **g**, except for the detrended HegyiDeng index. Note that the red time series in **c** and **d** are the 1.5N4-0.5N3 index and the HegyiDeng index with trend.

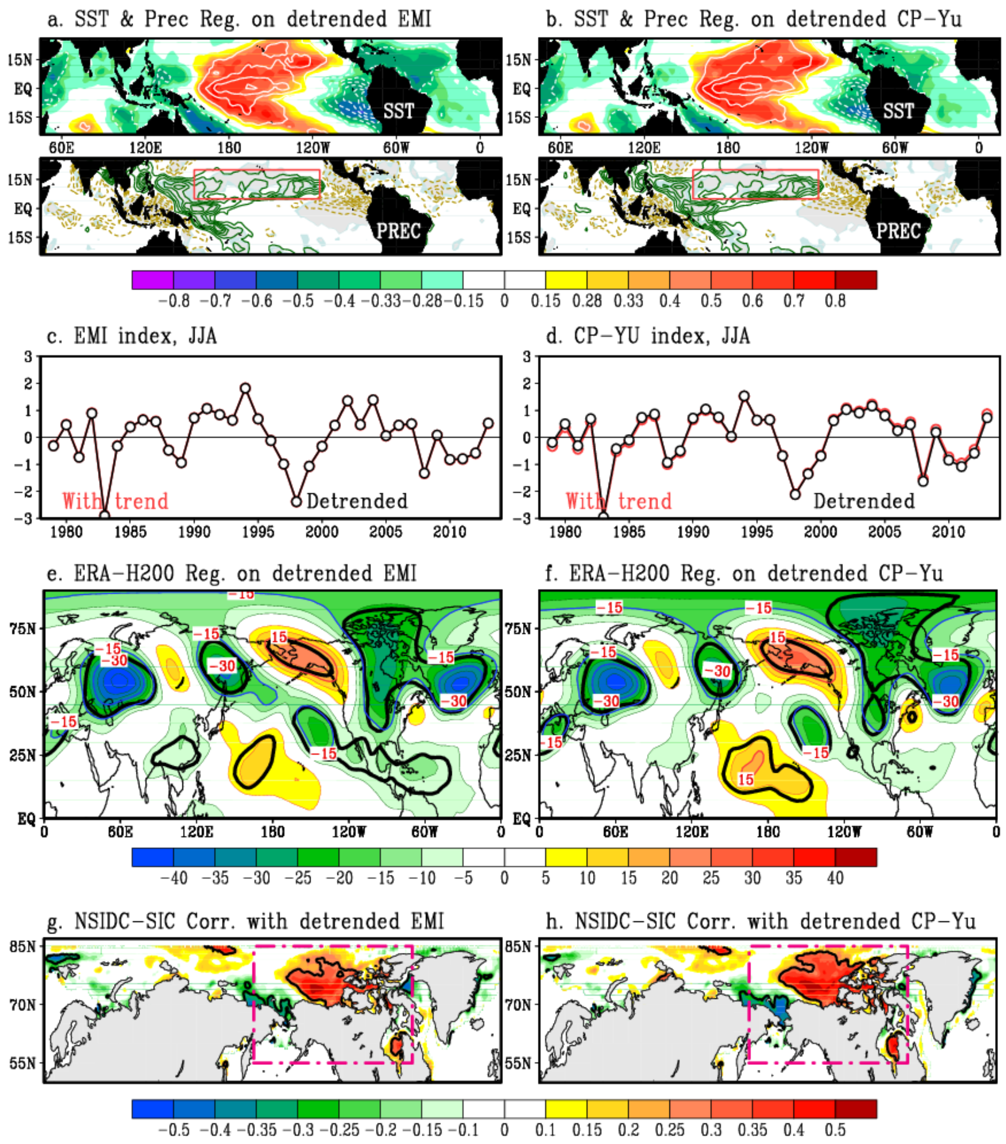

Supplementary Figure 15 | Statistics of detrended EMI and CP-Yu indices. Spatial patterns of tropical SST (**a**, top: color shadings are used for correlation, and contours are regression with an interval of 0.2°C), marine precipitation (**a**, bottom: regression with an interval of 0.4 mm day^{-1}), ERA-H200 (**e**, regression with an interval of 5 gpm) and NSIDC-SIC (**g**, correlation) associated with the detrended EMI index (**c**, in black). Gray shadings and thick black lines indicate the correlation at the estimated 90% confidence level. Panels **b**, **d**, **f** and **h** are the same as panels **a**, **c**, **e**, and **g** except for the detrended CP-Yu index. Note that the red time series in **c** and **d** are the EMI index and the CP-Yu index with trend.

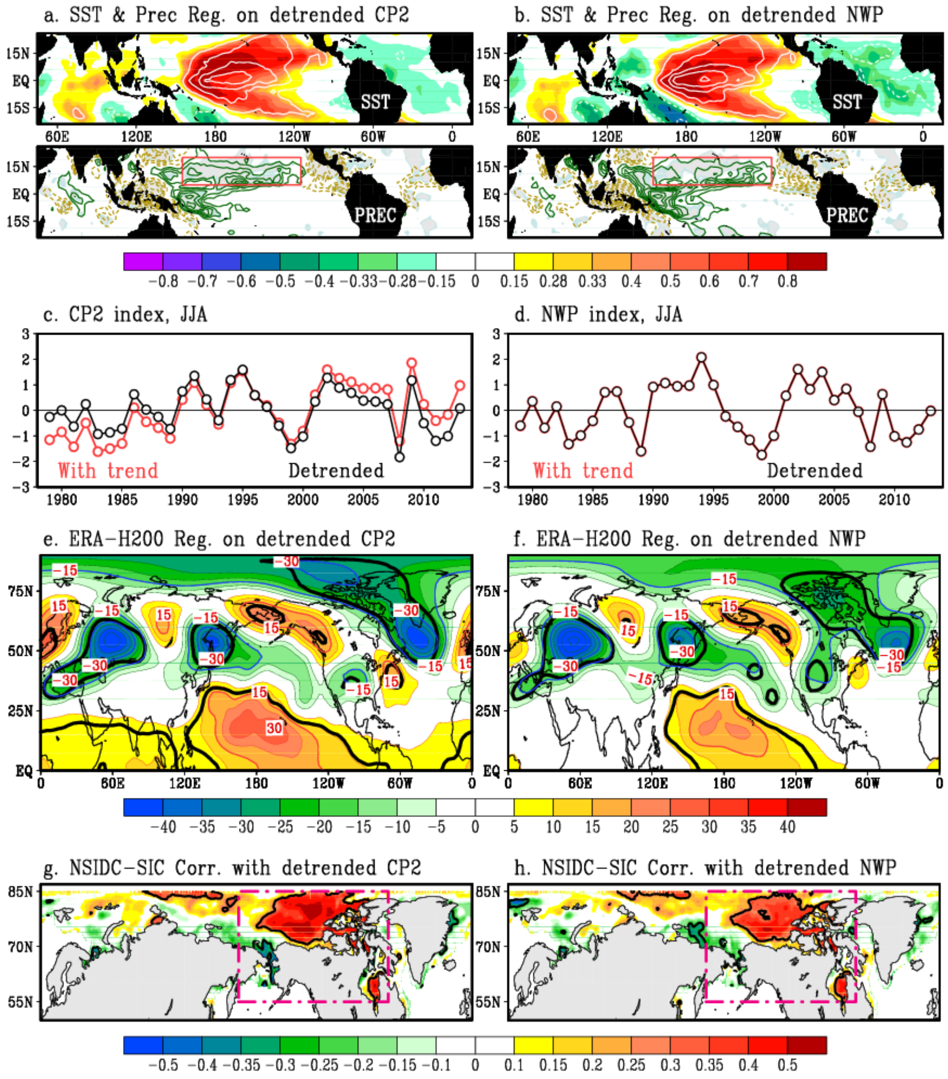

Supplementary Figure 16 | Statistics of detrended CP2-index and NWP index. Spatial patterns of tropical SST (**a**, top: color shadings are used for correlation, and contours are regression with an interval of 0.2°C), marine precipitation (**a**, bottom: regression with an interval of 0.4 mm day^{-1}), ERA-H200 (**e**, regression with an interval of 5 gpm) and NSIDC-SIC (**g**, correlation) associated with the detrended CP2-index (**c**, in black). Gray shadings and thick black lines indicate the correlation at the estimated 90% confidence level. Panels **b**, **d**, **f** and **h** are the same as panels **a**, **c**, **e** and **g**, except for the detrended NWP index. Note that the red time series in **c** and **d** are the CP2-index and the NWP index with trend.

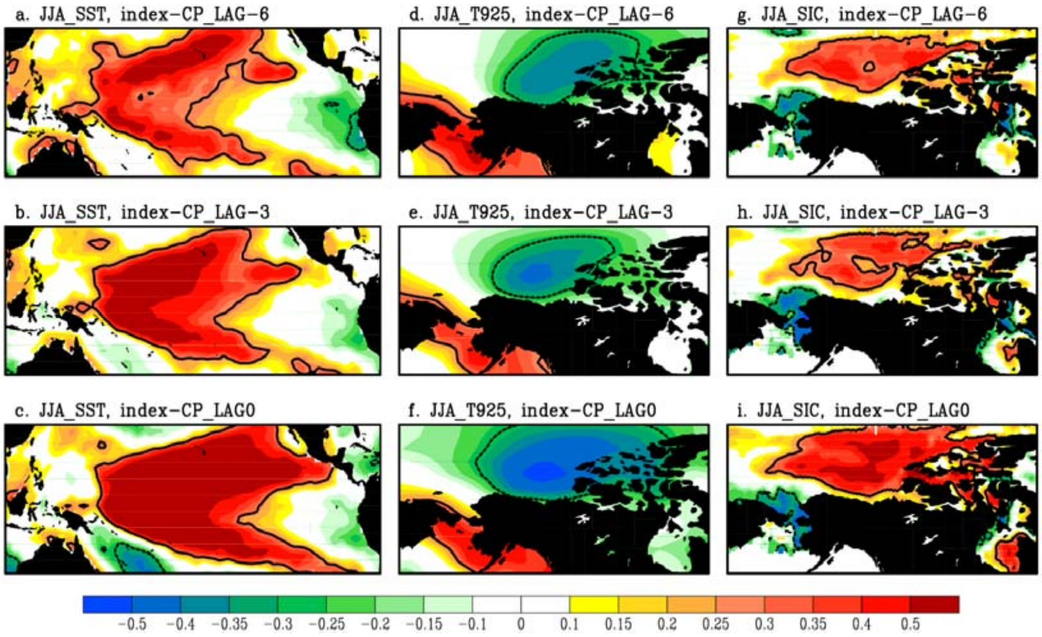

Supplementary Figure 17 | Lagged correlation maps. (**a**–**c**) Lagged correlations of summer tropical Pacific SST with the detrended CP index at lag(-6), lag(-3) and lag(0) months. Panels **d**–**f** and **g**–**i** are the same as **a**–**c**, except for ERA-T925 and NSIDC-SIC, respectively. Thick black lines indicate the correlation at the estimated 90% confidence level.

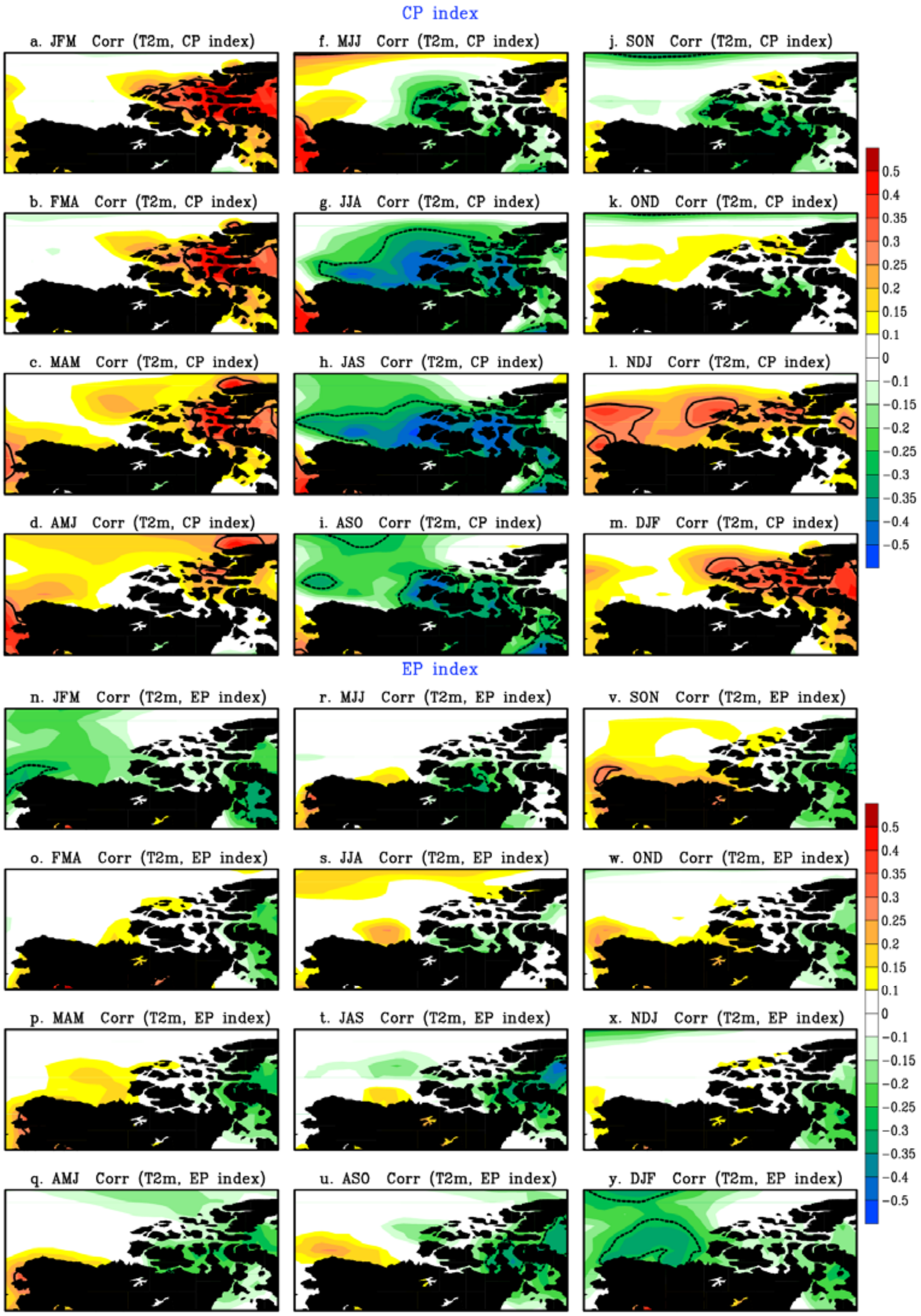

Supplementary Figure 18 | Teleconnection evolutions of T2m associated with CP index and EP index. (**a–m**) Correlations of ERA-T2m with the detrended CP index from JFM and FMA to DJF, respectively. Panels **n–y** are the same as **a–m**, except for the detrended EP index. Thick black lines indicate the correlation at the estimated 90% confidence level.

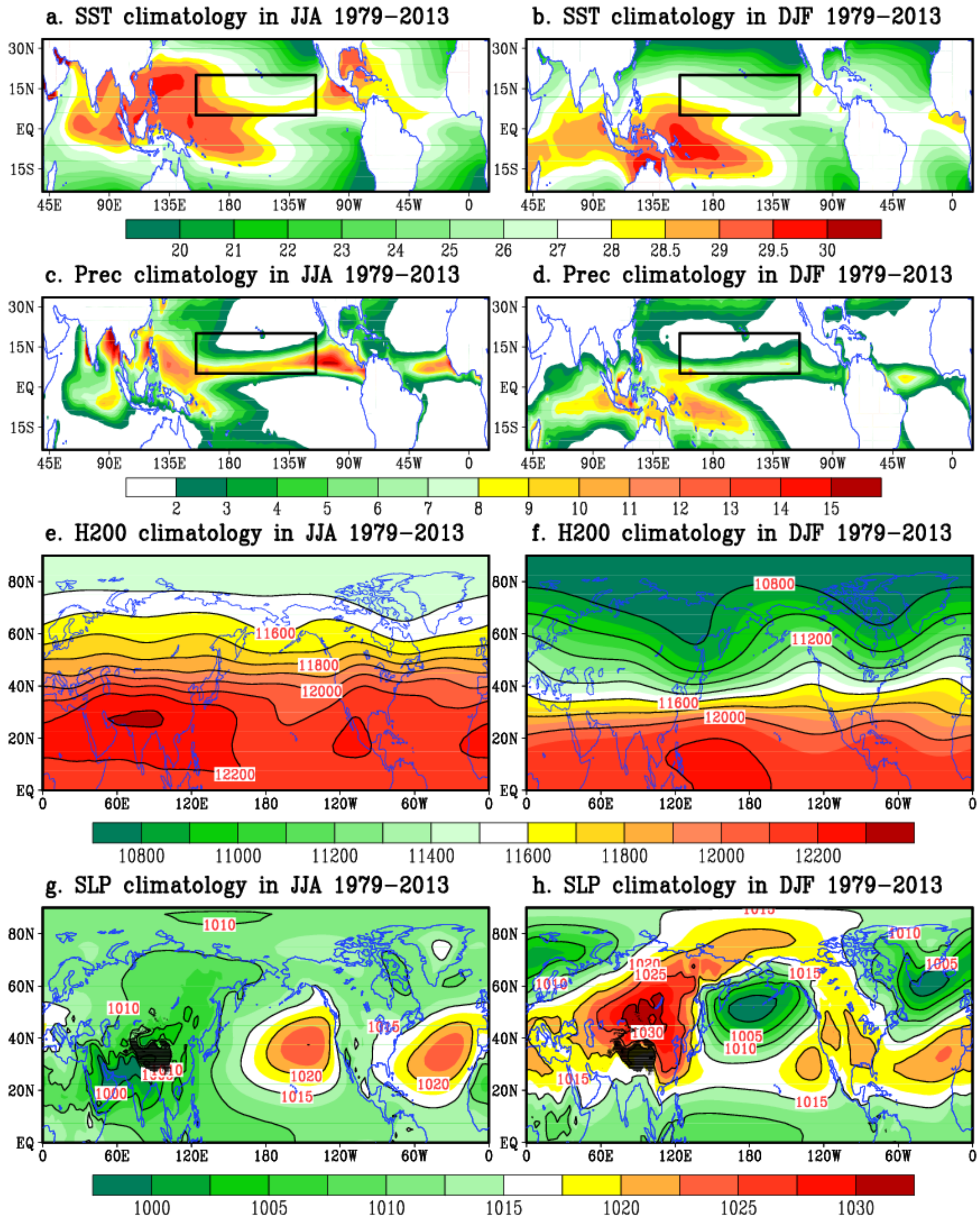

Supplementary Figure 19 | Summer and winter climatology background states for 1979–2013. Shown in **a–b**, **c–d**, **e–f** and **g–h** are respectively for SST, precipitation, ERA-H200, and ERA-SLP. Left panels are for summer (JJA), and right panels for winter (DJF). Black boxes outline the key forcing region (155°E–115°W, 5°N–20°N).

Supplementary Table 1 | Description of some ENSO-related index sources used in **Supplementary Figs. 13–16** (six CP El Niño indices are in bold type)

Index	Download Website or Source Calculations	Reference
Niño3	http://www.esrl.noaa.gov/psd/gcos_wgsp/Timeseries/Niño3/	Rayner et al. (ref. 11)
Niño4	http://www.esrl.noaa.gov/psd/gcos_wgsp/Timeseries/Niño4/	Rayner et al. (ref. 11)
NCT	NCT = Niño3 – a × Niño4, (if Niño3 × Niño4 >0 then a =2/5, else a =0)	Ren & Jin (ref. 6)
**NWP**	NWP = Niño4 – a × Niño3, (if Niño3 × Niño4 >0 then a =2/5, else a =0)	Ren & Jin (ref. 6)
**CP-Yu**	http://www.ess.uci.edu/~yu/2OSC/monthly_CP_index_1948_2014.txt	Yu & Kim (ref. 12)
Box.A of EMI	http://www.jamstec.go.jp/frcgc/research/d1/iod/DATA/emi.monthly.txt	Ashok et al. (ref. 13)
Box.B of EMI	http://www.jamstec.go.jp/frcgc/research/d1/iod/DATA/emi.monthly.txt	Ashok et al. (ref. 13)
Box.C of EMI	http://www.jamstec.go.jp/frcgc/research/d1/iod/DATA/emi.monthly.txt	Ashok et al. (ref. 13)
**EMI**	EMI = Box.A – 0.5 × Box.B – 0.5 × Box.C	Ashok et al. (ref. 13)
**HegyiDeng**	SST (165°E–130°W, 10°S–15°N)	Hegyi & Deng (ref. 14)
**1.5N4-0.5N3**	1.5 × Niño4 – 0.5 × Niño3	Garfinkel et al (ref. 1)
**CP2-index**	Same as the CP index, but for a smaller area (145°E–150°W, 16°S–16°N)	See **Methods**

Supplementary Table 2 | Correlation matrix of detrended CP and EP El Niño indices for 1979–2013; correlations > 0.43 are in bold type (99% confidence level)

Index	Niño3	NCT	1.5N4-0.5N3	EMI	CP-Yu	NWP	HegyiDeng	CP2-index
RPC1	**0.92**	**0.94**	-0.02	-0.30	-0.29	-0.04	**0.45**	-0.10
RPC2	0.23	0.08	**0.94**	**0.77**	**0.83**	**0.92**	**0.87**	**0.95**
EP index	**0.94**	**0.94**	0.12	-0.22	-0.16	0.10	**0.59**	0.12
CP index	0.12	-0.02	**0.87**	**0.71**	**0.80**	**0.85**	**0.76**	**0.99**
NCT	**0.98**	**1**	0.02	-0.23	-0.16	-0.02	**0.52**	0.01
NWP	0.15	-0.02	**0.98**	**0.88**	**0.85**	**1**	**0.82**	**0.86**

Supplementary Note 1

Capturing of the EP El Niño and CP El Niño modes

As suggested by Garfinkel et al. (ref. 1), “care must be taken when choosing the index used to identify central Pacific warming”. Accordingly, following Lian & Chen[2], in this study we use the REOF to capture the leading two modes (EP El Niño and CP El Niño) of the interannual variability of monthly normalized and detrended SST anomalies in the tropical Pacific for the following three reasons:

First, through a thorough statistical evaluation on the ability of non-rotated EOF and rotated EOF (REOF) in reproducing a large number of stationary modes, Lian & Chen[2] have demonstrated that REOF is overwhelmingly better than EOF in terms of accuracy and effectiveness, especially in capturing localized patterns of the EP El Niño and the CP El Niño.

Second, Trenberth & Stepaniak[3] suggested that SST normalization was desirable due to the differences in local SST variance.

Third, composite results support that there is no significant cold anomaly (the eastern part of El Niño Modoki) in the eastern Pacific along the coast of South America during CP El Niño[4-7]. Thus, non-rotated EOF decomposition has a tendency to produce unphysical modes due to its constraint of orthogonality in both space and time[2,8,9].

Supplementary Note 2

Robustness of CP warming-Arctic cooling teleconnection

There exist several different CP and EP El Niño definitions. Previous study has claimed that extratropical and stratospheric teleconnections are sensitive to the definitions chosen during boreal winter[1], but it has also been stated that analysis results are robust regardless of the CP El Niño definitions[10]. Although we have taken the advantage of REOF in capturing the different El Niño modes in **Supplementary Note 1**, we are also interested in the sensitivity of our results to different EP El Niño indices (i.e., Niño3 and NCT), especially the different CP El Niño indices (i.e., EMI, NWP, CP-Yu, etc.). These indices are listed in **Supplementary Table 1**, and their correlation matrix is shown in **Supplementary Table 2.** Relevant results are separately shown in **Supplementary Fig. 13** for two EP El Niño indices (Niño3 and NCT) and **Supplementary Figs. 14–16** for six CP El Niño indices (EMI, NWP, CP-Yu, 1.5N4-0.5N3, HegyiDeng, and CP2-index).

On the one hand, the features shown in **Supplementary Fig. 13** are almost the same as those obtained from the RPC1 and the EP index with no significant Arctic response. On the other hand, the **Supplementary Figs. 14–16** also mirror the same teleconnections obtained from the RPC2 and the CP index albeit with some changes in their significance. Nevertheless, it is at least suggested that our results derived from the REOF are not sensitive to the definitions chosen of the CP El Niño. Besides, at least three points are worthy to note:

First, Iza & Calvo (ref. 10) has pointed out that the HegyiDeng index is hybrid since the 1982/1983 and 1997/1998 EP El Niño cases were identified as the CP El Niño events by Garfinkel et al. (ref. 1). It is also seen from **Supplementary Table 2** that the HegyiDeng index is highly correlated with both EP and CP El Niño indices.

Second, although the H200 patterns associated with the other CP El Niño indices (**Supplementary Figs. 14–16**) are all consistent with those shown in **Fig. 1f**, the significances over both tropical regions and the Canada Basin become narrower and even insignificant compared to those shown in **Fig. 1f**. However, the Arctic SIC

patterns (**Supplementary Figs. 14–16**) are all significant as those shown in **Fig. 2c**, suggesting that the RPC2 derived from REOF are overwhelmingly better than other CP El Niño definitions, reconfirming previous theories/advantages about REOF, especially in capturing the two types of ENSO as claimed by Lian & Chen (ref. 2).

Third, considering that the definition of CP index is derived from the normalized SST anomalies at each grid and covers a larger spatial scale, the CP index can reflect more signals than the other CP ENSO indices. Therefore, the results obtained from the CP index are most robust. For example, when the CP index is replaced by the CP2-index (namely, when the definition area of CP index is narrowed to a smaller region for the CP2-index), the significance of the results becomes weaker over the Arctic region compared to **Supplementary Fig. 3f** and **Supplementary Fig. 16e**. However, both **Supplementary Fig. 3f** and **Supplementary Fig. 16e** are more significant than those obtained from the other five CP El Niño indices (i.e., EMI, NWP, CP-Yu, HegyiDeng, and 1.5N4-0.5N3). In addition, both the CP index and the CP2-index can reflect an obvious warming trend (**Supplementary Fig. 3d** and **Supplementary Fig. 16c**), whereas other five CP El Niño indices (i.e., EMI, NWP, CP-Yu, HegyiDeng, and 1.5N4-0.5N3) cannot reflect the trend. These features confirm that our definition contains more signals than other CP El Niño definitions because the SST normalization of each grid in a larger spatial scale is considered to define the CP index and the CP2-index.

Supplementary References

1. Garfinkel, C. I., Hurwitz, M. M., Waugh, D. W. & Butler, A. H. Are the teleconnections of central Pacific and eastern Pacific El Niño distinct in boreal wintertime? *Clim. Dyn.* **41,** 1835–1852 (2013).
2. Lian, T., & Chen, D. An evaluation of rotated EOF analysis and its application to tropical pacific SST variability. *J. Clim.* **25,** 5361–5373 (2012).
3. Trenberth, K. E. & Stepaniak, D. P. Indices of El Niño evolution. *J. Clim.* **14,** 1697–1701 (2001).
4. Yeh, S.-W., Kug, J.-S., Dewitte, B., Kwon, M.-H., Kirtman, B. P. & Jin F.-F. El Niño in a changing climate. *Nature* **461,** 511–514 (2009).
5. Kim, H.-M., Webster, P. J. & Curry, J. A. Impact of shifting patterns of Pacific Ocean warming on North Atlantic tropical cyclones. *Science* **325,** 77–80 (2009).
6. Ren, H.-L. & Jin, F.-F. Niño indices for two types of ENSO. *Geophys. Res. Lett.* **38,** L04704 (2011).
7. Sung, M.-K., Kim, B.-M. & An, S.-I. Altered atmospheric responses to eastern Pacific and central Pacific El Niños over the North Atlantic region due to stratospheric interference. *Clim. Dyn.* **42,** 159–170 (2014).
8. Horel, J. D. A rotated principal component analysis of the interannual variability of the Northern Hemisphere 500-mb height field. *Mon. Wea. Rev.* **109,** 2080–2092 (1981).
9. Richman, M. B. Rotation of principal components. *Int. J. Climatol.* **6,** 293–335 (1986).
10. Iza, M. & Calvo, N. Role of stratospheric sudden warmings on the response to central Pacific El Niño. *Geophys. Res. Lett.* **42,** 2482–2489 (2015).
11. Rayner, N. A. *et al.* Global analyses of sea surface temperature, sea ice, and night marine air temperature since the late nineteenth century. *J. Geophys. Res.* **108,** 4407 (2003).
12. Yu, J.-Y. & Kim, S. T. Identification of central-Pacific and eastern-Pacific types of ENSO in CMIP3 models. *Geophys. Res. Lett.* **37,** L15705 (2010).
13. Ashok, K., Behera, S. K., Rao, S. A., Weng, H. & Yamagata, T. El Niño Modoki and its possible teleconnection. *J. Geophys. Res.* **112,** C11007 (2007).
14. Hegyi, B. M. & Deng, Y. A dynamical fingerprint of tropical Pacific sea surface temperatures on the decadal-scale variability of cool-season Arctic precipitation. *J. Geophys. Res.* **116,** D20121 (2011).
15. Plumb, R. A. On the three-dimensional propagation of stationary waves. *J. Atmos. Sci.* **42,** 217–229 (1985).
16. Takaya, K. & Nakamura, H. A formulation of a phase-independent wave-activity flux for stationary and migratory quasigeostrophic eddies on a zonally varying basic flow. *J. Atmos. Sci.* **58,** 608–627 (2001).
